# Loss of linker histone H1 in the maternal genome influences DEMETER-mediated demethylation and affects the endosperm DNA methylation landscape

**DOI:** 10.3389/fpls.2022.1070397

**Published:** 2022-12-22

**Authors:** Qiang Han, Yu-Hung Hung, Changqing Zhang, Arthur Bartels, Matthew Rea, Hanwen Yang, Christine Park, Xiang-Qian Zhang, Robert L. Fischer, Wenyan Xiao, Tzung-Fu Hsieh

**Affiliations:** ^1^ Department of Biology, Saint Louis University, St. Louis, MO, United States; ^2^ Department of Plant and Microbial Biology, North Carolina State University, Raleigh, NC, United States; ^3^ Plants for Human Health Institute, North Carolina State University, Kannapolis, NC, United States; ^4^ College of Food Science and Engineering, Foshan University, Foshan, China; ^5^ Department of Plant and Microbial Biology, University of California, Berkeley, Berkeley, CA, United States

**Keywords:** epigenetics, DNA demethylation, gene imprinting, DNA glycosylase, linker histone H1

## Abstract

The *Arabidopsis* DEMETER (DME) DNA glycosylase demethylates the central cell genome prior to fertilization. This epigenetic reconfiguration of the female gamete companion cell establishes gene imprinting in the endosperm and is essential for seed viability. DME demethylates small and genic-flanking transposons as well as intergenic and heterochromatin sequences, but how DME is recruited to these loci remains unknown. H1.2 was identified as a DME-interacting protein in a yeast two-hybrid screen, and maternal genome H1 loss affects DNA methylation and expression of selected imprinted genes in the endosperm. Yet, the extent to which H1 influences DME demethylation and gene imprinting in the *Arabidopsis* endosperm has not been investigated. Here, we showed that without the maternal linker histones, DME-mediated demethylation is facilitated, particularly in the heterochromatin regions, indicating that H1-bound heterochromatins are barriers for DME demethylation. Loss of H1 in the maternal genome has a very limited effect on gene transcription or gene imprinting regulation in the endosperm; however, it variably influences euchromatin TE methylation and causes a slight hypermethylation and a reduced expression in selected imprinted genes. We conclude that loss of maternal H1 indirectly influences DME-mediated demethylation and endosperm DNA methylation landscape but does not appear to affect endosperm gene transcription and overall imprinting regulation.

## Introduction

DNA methylation regulates important processes in eukaryotic genomes including gene transcription, transposon silencing and genomic imprinting (Law and Jacobsen, 2010). In plants, *de novo* methylation is guided by small interfering RNAs in a process known as the RNA-directed DNA methylation (RdDM) (Matzke and Mosher, 2014). Once DNA methylation is established, it is maintained upon replication by distinct DNA methyltransferases to preserve cell identity and genome integrity. Plant DNA methylation is found in CG, CHG, and CHH sequence contexts (H is A, C, or T) and is mainly targeted to transposons or repetitive sequences of the genome. DNA methylation also needs to be dynamically reconfigured during development to enable transition to a new cell fate and transcriptional state. Such epigenetic reconfiguration plays a prominent role in animal reproduction and is required for reproductive success in flowering plant (Ono and Kinoshita, 2021). Removal of DNA methylation in plant is catalyzed by the DNA glycosylase DEMETER (DME), repressor of silencing1 (ROS1), DEMETER LIKE2 (DML2), and DML3 in *Arabidopsis* (Agius et al., 2006; Gehring et al., 2006) through a Base Excision Repair (BER) pathway. Whereas ROS1, DML2 and DML3 are more widely expressed and function to counteract the spread of RdDM methylation into nearby coding genes, DME demethylation during reproduction establishes gene imprinting in the endosperm and is essential for seed viability (Choi et al., 2002; Gehring et al., 2006; Gent et al., 2022; Xu et al., 2022).

During *Arabidopsis* reproduction, the central cell genome is extensively demethylated at about nine thousand loci prior to fertilization. This epigenetic reconfiguration differentiates the imprints of parental genomes and establishes the parent-of-origin specific expression of many imprinted genes, including two essential components of the PRC2 complex (i.e., *MEDEA* and *FIS2*) crucial for seed development (Choi et al., 2002; Jullien et al., 2006; Huh et al., 2008). In pollen, DME also demethylates the vegetative cell genome to reinforce DNA methylation of the sperm and to ensure a robust pollen germination in certain ecotypes (Schoft et al., 2011; Ibarra et al., 2012). Genomic regions hypermethylated in *dme* mutant reside primarily in gene-flanking small euchromatic transposons as well as in many intergenic and heterochromatin sequences. Such loci that satisfy differential methylation criteria were generally referred to as DME target loci, even though a direct physical DME localization remains to be demonstrated. (Ibarra et al., 2012). How DME is recruited to distinct genomic regions with different chromatin states remains elusive, although the Facilitates Chromatin Transactions (FACT) histone chaperone is required for demethylation of heterochromatin and certain imprinted loci in the central cell (Ikeda et al., 2011; Frost et al., 2018). FACT complex is known to play a pivotal role in almost all chromatin-related processes, including transcription, replication, and DNA repair (Hondele and Ladurner, 2013). This is because nucleosomes are barriers to these processes that require access to the nucleosomal DNA, and the FACT complex is needed to facilitate destabilizing and disassembling nucleosomes. Interestingly, the FACT complex is only required for DME demethylation in central cell but not in vegetative nuclei (Frost et al., 2018), highlighting a difference in their chromatin conformation. There are three canonical histone H1 variants in Arabidopsis, the ubiquitous H1.1 and H1.2 variants and the stress-inducible H1.3 (Gantt and Lenvik, 1991; Ascenzi and Gantt, 1999a; Wierzbicki and Jerzmanowski, 2005; Kotlinski et al., 2017). The vegetative cell has a decondensed nuclei and highly dispersed heterochromatin (Schoft et al., 2009); the two canonical linker histone H1.1 and H1.2 are specifically absent in the vegetative cell, which might contribute to the differential requirement of FACT for DME function (Hsieh et al., 2016; He et al., 2019).

H1 linker histones are conserved eukaryotic nuclear proteins required to maintain higher order chromatin structure and DNA methylation patterns in plants and animals (Fan et al., 2005; Wierzbicki and Jerzmanowski, 2005; Zemach et al., 2013). H1 is found more enriched in heterochromatin than euchromatin in Arabidopsis and is thought to participate in heterochromatin condensation in plant cells (Ascenzi and Gantt, 1999b; Rutowicz et al., 2015; Choi et al., 2020). H1 loss causes dispersion of heterochromatin, nucleosome reorganization, and de-repression of H1-bound genes (Rutowicz et al., 2019; Choi et al., 2020). It has been suggested that H1-enriched chromatins are less accessible to DNA modifying enzymes, which is supported by the observations that gain in heterochromatin DNA methylation due to H1 loss likely results from increased access by DNA methyltransferases to the less condensed heterochromatin (Zemach et al., 2013; Lyons and Zilberman, 2017). However, euchromatic TEs exhibit a loss in DNA methylation in *h1* double mutant (*h1.1-1*,*h1.2-1*), for reasons not fully understood (Zemach et al., 2013). Although earlier studies have shown that reducing the level of histone H1 affected DNA methylation patterns of some imprinted genes in mammals and plants (Fan et al., 2005; Wierzbicki and Jerzmanowski, 2005; Rea et al., 2012), how H1 takes part in these processes has not been elucidated.

H1.2 interacts with DME in a yeast two-hybrid screen and an *in vitro* pull-down assay and its isoforms H1.1 and H1.3 were also shown to interact with DME (Rea et al., 2012). In the endosperm derived from a cross between female *h1* triple mutant (*h1.1, h1.2-1, h1.3*) and wild-type pollen, the expression of selected DME-regulated imprinted genes (*MEA*, *FIS2*, *FWA*) was reduced, accompanied with an increase in the methylation of their maternal alleles, suggesting that H1 might play a role in mediating DME demethylation. However, loss of H1 differentially influences DNA methylation in heterochromatic and euchromatic TEs, raising the possibility that these effects might be epistatic to DME action. Here we showed that both *H1.1* and *H1.2* promoters are active in the central cell, with *H1.1* being more ubiquitously expressed in ovule while *H1.2* is preferentially expressed in the central cell. Our methylome analysis showed that H1 loss in the maternal genome resulted in endosperm heterochromatin hypomethylation, suggesting that the central cell chromatin is less condensed and more accessible by DME in the absence of H1. Maternal H1 loss differentially affected methylation pattern of canonical DME DMRs but did not substantially affect parent-of-origin specific expression of a list of reported imprinted genes. Our results revealed that loss of H1 indirectly affects DME-mediated DNA demethylation in the central cell and endosperm genome methylation but does not alter overall gene transcription or imprinting regulation in the endosperm.

## Materials and methods

### Plant materials and growth conditions

*Arabidopsis* plants were grown on soil in growth chambers under 16h, 23°C day/8h, 22°C night growth condition. *h1* mutant T-DNA insertional lines were obtained from ABRC and used to generate *h1* higher order mutants. *h1.1-1* (SALK_128430C) has a T-DNA insertion in the first exon, at 133bp downstream of the start codon. *h1.2-1* (Salk_002142) has a T-DNA insertion in the promoter region and the h*1.3-1* (SALK_025209) has a T-DNA insertion also in the promoter region, 62bp upstream of the transcription start. *H1.2* expression was still detectable in the *h1.2-1* allele. We therefore searched for another stronger *h1.2* T-DNA line and found CS438975 (we named as *h1.2-2*), which has a T-DNA insertion in the first exon, at 115 bp downstream of the ATG. Each *h1* single mutant was backcrossed to wild type for 4-6 time before analysis and used for generating higher order mutants. Expression of *H1.2* was undetectable in the *h1.2-2* mutant allele. This *h1.2-2* allele has since been used in multiple studies (Zemach et al., 2013; Lyons and Zilberman, 2017; He et al., 2019; Choi et al., 2020; Choi et al., 2021; Bourguet et al., 2022).

### GUS reporter constructs

The promoter region of histone *H1.1* was PCR amplified with primers H1.1promoter_HindIII_fwd (5’-CCCAAGCTTAAGATGTTTTAGATTGATTT-3’) and H1.1promoter_BamHI_rev (5’-CGCGGATCCCGTCTTCTGAACTTAAGATC-3’). The promoter region of histone *H1.2* was PCR amplified with primers H1.2promoter_SalI_fwd2 (5’-ACGCGTCGACGGTTAGATTTTGAATTGGAA-3’) and H1.2promoter_XbaI_rev2 (5’-TGCTCTAGACTTCTTCTCTCTCAGAAACT-3’). The promoter region of histone *H1.3* was PCR amplified with primers H1.3promoter_HindIII_fwd (5’-CCCAAGCTTAGAGTTTTAGCTTAGTTTTA-3’) and H1.3promoter_BamHI_rev (5’-CGCGGATCCTAGAGGATTAGTGAAAGTGT-3’). The amplified *H1* promoter regions were fused with reporter gene GUS (β-glucuronidase) in the binary vector pBI101, respectively. The constructs were confirmed by sequencing and transformed into *Arabidopsis* Col-0 by *Agrobacterium* infiltration. After screening for transgenic plants, stable transgenic T2 or later plants were used for GUS staining analysis. Tissue samples from young seedlings, different stages of floral buds and seeds were harvested and put immediately into the GUS staining buffer (10 mM sodium phosphate buffer, pH 7.2, 0.5% Triton X-100, and 1 mg/mL 5-bromo-4-chloro-3-indolyl- β-glucuronic acid) on ice. Samples in the staining buffer were vacuum infiltrated on ice for 30 minutes. Samples were incubated at 37°C with rotation for 18 hours or less. Tissues were destained using 30%, 50%, 70% ethanol in sequence for 30 minutes each time with rotation. Samples were imbibed in seed clearing solution or diH_2_O on slides for imaging.

### GFP reporter constructs

**Figure 1:**
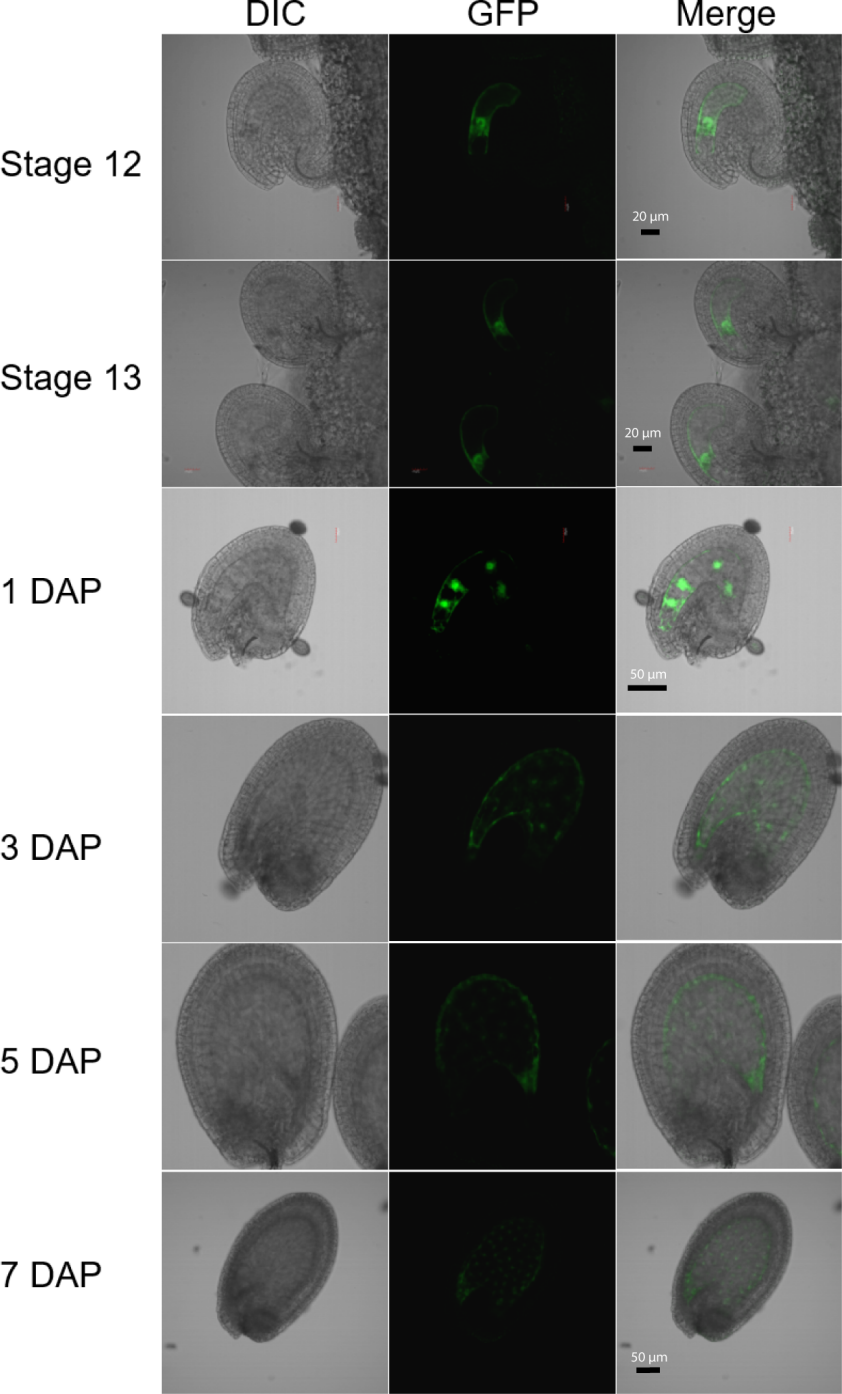
Expression of the *H1.2 promoter::GFP* transgene in *Arabidopsis* ovules and young developing seeds. Ovules and seeds with expression of *H1.2 promoter*:*GFP* transgene were photographed using confocal fluorescence microscope. Ovules at flower stage 12 and stage 13 and seeds at 1, 3, 5, and 7 DAP were hand-dissected for imaging. The GFP signal is shown in green. DAP, Days After Pollination; DIC, Differential Interference Contrast. Scale bars: State 12 and 13, bar = 20 um; 1-5 DAP, bar = 50 um; 7 DAP, bar = 50 um.

The *H1.1* promoter region (the 1296-bp genomic DNA of the *H1.1* promoter sequence starting from the end of 3’UTR of the upstream gene to the end of 5’UTR of *H1.1*) was PCR amplified with primers H1.1pro_SalI_fwd (5’- ACG CGT CGA CAA GAT GTT TTA GAT TGA TTT -3”) and H1.1pro_SalI_rev (5’- ACG CGT CGA CCG TCT TCT GAA CTT AAG ATC-3’). The *H1.2* promoter region (the 2036-bp genomic DNA including 1607-bp upstream of transcription start site of *H1.2* plus 429-bp 5’UTR of *H1.2*) was PCR amplified with primers His1.2 5’ SalI (5’- ACGCGTCGACTGGTTCGAGTATTTTA-3’) and His1.2 3’ XbalI (5’- GCTCTAGACTTCTTCTCTCTCAGAAA-3’). The *H1.3* promoter region (the 1279-bp genomic DNA of the *H1.3* promoter sequence starting from the end of 3’UTR of the upstream gene to the end of 5’UTR of *H1.3*) was PCR amplified with primers H1.3proSalIfwd (5’- ACG CGT CGA CAG AGT TTT AGC TTA GTT TTA AAA ATC -3’) and H1.3proSalIrev (5’- ACG CGT CGA CTA GAG GAT TAG TGA AAG TGT-3’). The amplified *H1* promoter regions were cloned into vector pBI-EGFP. These transcriptional fusion reporter constructs were confirmed by sequencing and transformed into *Arabidopsis* Col-0 by *Agrobacterium* infiltration. After screening for transgenic plants, stable transgenic T2 or later plants were used for GFP expression analysis. Flowers before and after fertilization were harvested. Ovules at flower stage 12-13 and seeds were dissected out and imbibed in diH_2_O on slides for imaging using confocal microscopy. GFP was excited by the 488 nm laser line and was detected in a range between 505 nm to 535 nm. Approximately 40 stable transgenic lines were obtained and screened, 4-6 GUS reporter lines and 3-5 GFP reporter lines for each promoter construct were examined and representative expression pattern were reported in **Figures 1**, **S1**–**S3**.

**Figure 2:**
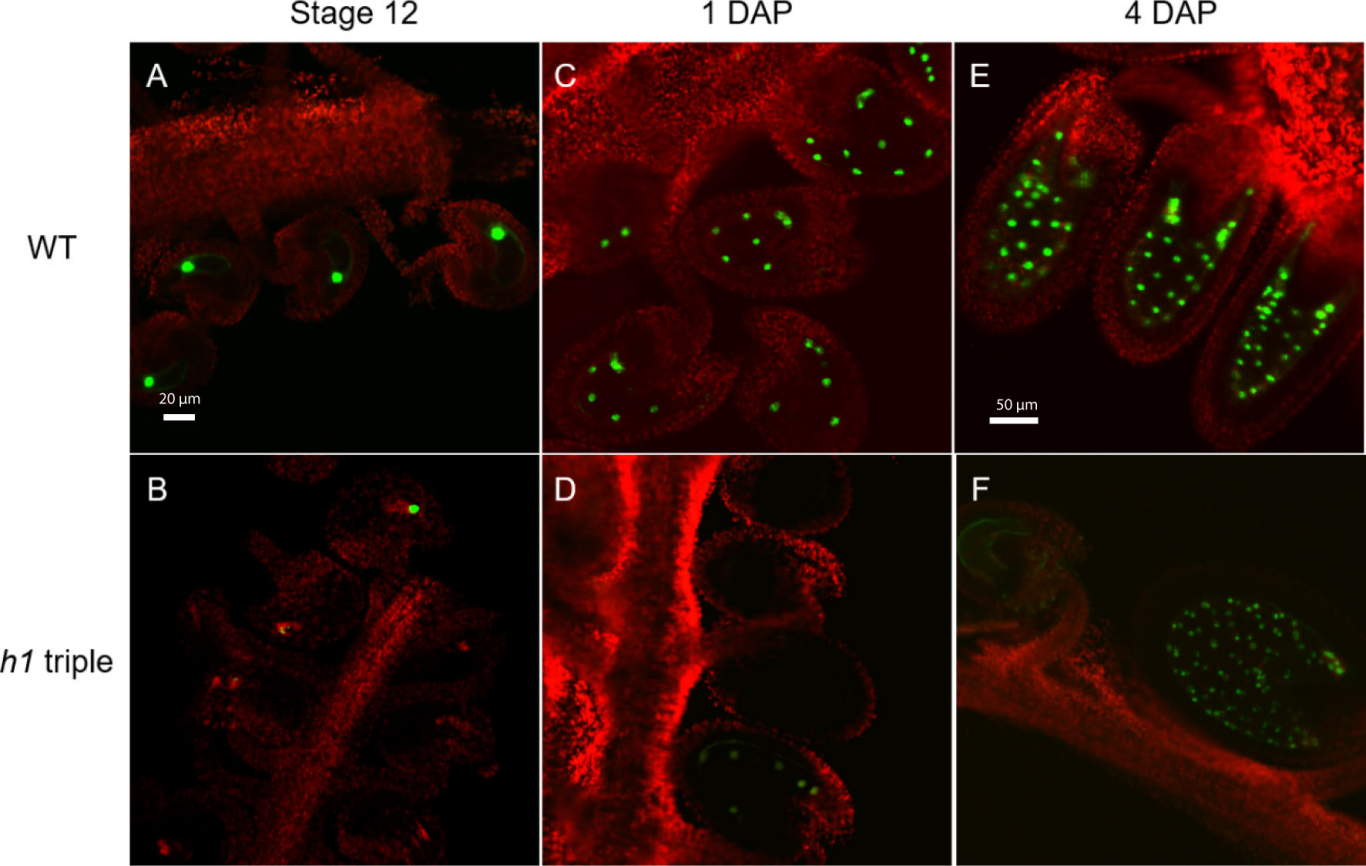
Expression of the *H1.2 promoter::GFP* transgene is reduced in the central cell of h*1* hybrid endosperm. Fluorescence images of *pMEA::GFP* expression signals in wild type **(A, C, E)** and in *h1* triple mutant endosperm **(B, D, F)**. The GFP and chlorophyll fluorescence signals were pseudo-colored as green and red, respectively. Fluorescence micrographs of ovules at flower stage 12 **(A, B)**, 24-hr after pollination **(C, D)**, and 96-hr after pollination **(E, F)**.

The *pFWA*::GFP construct was a gift from Dr. Tetsu Kinoshita (Yokohama City University) and was the same construct used in (Ikeda et al., 2011). The *MEDEA* promoter-GFP construct contains a 4.238 bp of *MEA* promoter amplified with pMEAF (5’-AGACGGACGTCCTGACGCTAACGTCCTGTCAAACCCGTCCCGTAA-3’) and pMEAR (5’-TCTGCCTTCGCCATTAACCACTCGCCTCTTCTTTTTTTCTC -3’), an Arabidopsis H2B (AT5G22880) fused, nuclear-localized GFP amplified with meaHTB2F (5’-AGTGGTTAATGGCGAAGGCAGATAAGAAACCA-3’) and GFPmeaR (5’-GCTGCTTCTCCTCAGATCAAAAATTACTTGTACAGCTCGTCCATGC-3’) from pBIn1GFP plasmid (Zhang et al., 2008), a 2.1 kb *MEDEA* 3’-end sequence amplified with 3meaF (5’-TTTTTGATCTGAGGAGAAGCAGCAATTCAAGCA-3’) and 3meaR (5’-ACTCTAGGGACTAGTCCCGGGTTTCATATTCTTGATTCGCCAAATCAGTG-3’). The 3 fragments were concatenated using the Gibson assembly method. The backbone plasmid is a binary plasmid vector, pFGAMh (a hygromycin resistant version of pFGC5941) described before (Zhang et al., 2019). Both constructs were transformed into wild type and the *h1* triple homozygous mutant *Arabidopsis* plants, respectively. The transgenic plants were analyzed for GFP expression in ovules and seeds. For *pMEA::GFP*, we obtained and analyzed 8 independent lines in wild type and 7 independent lines in *h1* triple mutant. For *pFWA::GFP*, 12 and 7 independent lines were obtained and analyzed in wild-type Col-0 and in *h1* triple mutant, respectively. Representative images are presented in **Figures 2**, **S5**.

### Purification of endosperm nuclei through fluorescence activated cell sorting (FASC)

Embryo and endosperm from 7 - 8 DAP seeds were dissected under a dissecting microscope. For DNA extraction, embryo and endosperm were collected in a tube with 140 ul nuclei extraction buffer and protease inhibitor as described (Zheng and Gehring, 2019), and ground with a pestle on ice. Each sample was ground for 2 minutes, then 800ul nuclei staining buffer was added. Tubes containing nuclei staining buffer were wrapped with aluminum foil to avoid light. The nuclei in extraction buffer and staining buffer were filtered twice with CellTrics 30 um filter on ice, and kept on ice until FASC. The nuclei were sorted at the Flow Cytometry Research Core Facility at Doisy Research Center, Saint Louis University, using a BD FACSAriaI IIu equipped with a 407 nm violet laser using the following parameters: Nozzle size: 100 microns; Sheath pressure: 30 psi; Droplet frequency: 28,100 drops/sec.; Precision mode: Purity. Nuclei were gated based on signals from the DAPI channel.

For RNA extraction, embryos and endosperms were dissected and put into 1.5 ml Eppendorf tubes floated on liquid nitrogen. After collection, embryos and endosperms are kept in -80°C. Nuclei extraction buffer and nuclei staining buffer are added before cell sorting. After sorting, purified embryo and endosperm nuclei were collected with 1.5 ml Eppendorf tube in 50 ml TRIzol™ Reagent (Invitrogen), Zymo RNA Lysis buffer or other RNA lysis buffer which were used for RNA extraction immediately after sorting.

### Whole-genome bisulfite sequencing and DNA methylome analysis

Crosses were performed between *h1* triple mutant *h1.1-1 h1.2-2 h1.3-1* (female) and wild type L*er* (male). Embryos and endosperms were collected 7 - 8 days after crossing and desired nuclei fractions purified through FACS. Genomic DNA was isolated from FACS-purified nuclei using CTAB method as described (Ibarra et al., 2012). Approximately 2-5 ng of purified genomic DNA was spiked with 1% (w/w) of unmethylated cl857 *Sam7* Lambda DNA (Promega, Madison, WI) and sheared to about 400bp using Covaris M220 (Covaris Inc., Woburn, Massachusetts) under the following settings: target BP, 350; peak incident power, 75 W; duty factor, 10%; cycles per burst, 200; treatment time, 60 second; sample volume 50μl. The sheared DNA was cleaned up and recovered by 1.2x AMPure XP beads followed by one round of sodium bisulfite conversion using the EZ DNA Methylation-Lightning Kit (Zymo Research Corporation, Irvine, CA) as outlined in the manufacturer’s instruction with 80 min of conversion time. Bisulfite sequencing libraries were constructed using the ACCEL-NGS Methyl-Seq DNA library kit (Product Code 30024, Swift Biosciences, Ann Arbor, MI) per manufacturer’s instruction. The PCR enriched libraries were purified twice with 0.8x (v/v) AMPure XP beads to remove adaptor dimers. High throughput sequencing was performed by Novogene Corporation (Davis, CA.). Sequencing reads from three individual transgenic lines were used in the analysis (**Table S1**). Sequenced reads were mapped to the TAIR10 (whole genome) and TAIR8 (allele-specific) reference genomes and DNA methylation analyses were performed as previously described (Ibarra et al., 2012).

Fractional CG methylation in 50-bp windows across the genome was compared between embryo and endosperm from *h1/H1* seeds as well as wild-type embryo, endosperm, and *dme-2* endosperm (GSE38935). Windows with a fractional CG methylation difference of at least 0.3 (Fisher’s exact test p-value < 0.001) were merged to generate larger differentially methylated regions (DMRs) if they occurred within 300 bp. Merged DMRs were retained for further analysis if the fractional CG methylation difference across the merged DMR > 0.3 (Fisher’s exact test *p-*value < 10-6), and if the DMR is at least 100-bp long. The merged DMR list is in the **Supplemental Dataset S1**. Distribution of DMRs along genes and whole genome methylation average metaplots of genes and TEs were plotted as described previously (Ibarra et al., 2012; Zhang et al., 2019). DNA methylation kernel density plots compare fractional methylation within 50-bp windows. We used windows with at least 10 informative sequenced cytosines and fractional methylation of at least 0.5 in at least one of the samples being compared as described before (Ibarra et al., 2012; Zhang et al., 2019).

### RNA sequencing

Embryos and endosperms were collected 8 days after cross and total RNA was extracted using RNAeasy kit (Qiagen) plus on-column DNase I digestion. Three independent sets of total RNAs from embryo and endosperm of *h1* Col x L*er* and wild-type Col x L*er* were isolated. Illumina cDNA libraries were constructed with the Ovation RNA-seq System V2 (NuGen Technologies) per manufacturer’s instruction and as described (Hsieh et al., 2011). Adapter trimming was performed using the fastp program (Chen et al., 2018). For allele-specific transcriptome analysis, adapter-trimmed sequencing reads were mapped to the TAIR8 Col-0 and L*er* cDNA scaffold using custom scripts as before (Hsieh et al., 2011). Individual reads were assigned to Col-0 or L*er* ecotype based on the SNPs detected between the two ecotypes. Each gene received Col-0 and L*er* scores, which represented the number of reads aligned to the corresponding ecotype. These numbers were used to calculate the maternal transcript proportion for each gene. For endosperm and embryo transcriptomes, adapter-trimmed sequencing reads were aligned to TAIR10 for transcript assignation and quantification with Salmon (v.1.9.0) (Patro et al., 2017). DESeq2 was used to identify differentially expressed genes (Love et al., 2014).

## Results

### Histone *H1.2* promoter is active in the central cell and developing endosperm

Although DME is preferentially active in the central cell, it is also expressed in the pollen vegetative cell (VC) and demethylates the VC genome during *Arabidopsis* reproduction (Schoft et al., 2011; Ibarra et al., 2012). However, H1.1 and H1.2 are present in sperm but undetectable in vegetative nuclei and *H1.3* expression is not detected in pollen (Hsieh et al., 2016; He et al., 2019). This indicates that H1 is not required for DME demethylation in VC. To examine whether histone *H1* is expressed in central cell, we fused the three *H1* genes’ promoter regions with the *GFP* reporter gene respectively and transferred these transcriptional GFP reporter constructs into *Arabidopsis* Col-0. Although both *H1.1* and *H1.2* were reported to be widely expressed (Rutowicz et al., 2015), we were surprised to see that the *H1.2* promoter was preferentially active in the central cell of stage 12 flowers but undetectable during earlier gametogenesis (**Figure 1**). After fertilization, *H1.2* expression is observed in the nuclear cytoplasmic domain (NCD) of early endosperm, but the expression level decreases as endosperm develops (**Figure 1**). *H1.2* expression was not visible in embryo although very weak GFP signals sometimes can be seen in the septum, funiculus, and integuments (**Figure S1**). The *H1.1* promoter-GFP construct was more ubiquitously expressed in ovules and seeds including the integument, central cell, embryo, and endosperm (**Figure S2**). The *H1.3* promoter-GFP was generally not expressed but GFP signals were visible around incision areas and in the septum. These signals are likely artifactually induced during dissecting of ovules because *H1.3* expression was stress inducible (Rutowicz et al., 2015) and *H1.3* promoter-GUS did not show similar expression pattern (**Figure S3**).

The same set of *H1* promoters driving the GUS reporter genes confirmed what was reported that both *H1.1* and *H1.2* are widely expressed, with *H1.1* being more abundantly expressed than *H1.2* whereas *H1.3* is very lowly expressed in the tissues assessed (**Figure S3**). Gamete expression of *H1.2* has been reported by Song et al. with an average expression of 135.86 (TPM normalized) in the central cell which is about 15-fold higher than the average expression of 9.26 in the egg cell (Song et al., 2020). After fertilization, however, the transcripts of *H1.1* and *H1.2*, but not those of *H1.3*, can be detected from pre-globular embryo proper through mature embryo, with *H1.1* being expressed at 2x - 4x more abundantly than *H1.2*, according to the Gene Networks in Seed Development database (**Figure S4**) (Belmonte et al., 2013).

Central cell-preferred expression of *H1.2* in the mature ovule was unexpected. We wondered whether *H1.2* expression in the central cell is regulated by *DME*. To test this, we crossed the homozygous *pH1.2:GFP* transgenic plants with *DME/dme-2* heterozygotes and obtained the F1 plants that were heterozygous for *DME/dme-2* and hemizygous for the *pH1.2:GFP* transgene. In the F2 generation, we examined the *pH1.2:GFP* expression in the *DME/dme-2* mutant and their segregating wild-type siblings. We observed that 48% of the female gametophytes with ovules showed strong GFP signal in the *DME/DME pH1.2:GFP*/- (87 ovules expressed GFP out of 183 total ovules, 87:96, 1:1, χ2 = 0.44, P > 0.50). and 47% of expression in the *DME/dme*-2 *pH1.2:GFP*/- plants (83 ovules expressed GFP out of 176 total ovules, 83:93, 1:1, χ2 = 0.56, P > 0.46). This result indicates that *H1.2* expression in the central cell is not regulated by *DME*. This is consistent with *H1.2* not being an imprinted gene as we reported earlier (Rea et al., 2012).

### Loss of histone H1 affects the expression of *MEA* and *FWA* before and after fertilization

We previously showed that in the endosperm derived from *h1* x L*er* cross (referred to as *h1/H1* cross thereinafter), the expression of *MEA* and *FWA* was reduced (Rea et al., 2012). To gain a better understanding on how loss of H1 affects *MEA* and *FWA* expression, we used *MEA* and *FWA* promoter::*GFP* constructs and transferred them into *Arabidopsis* wild type Col-0 and the *h1* triple mutant. Transgenic plants homozygous for *pMEA::GFP* and *pFWA::GFP* were used to examine *MEA* and *FWA* gene expression. In wild-type plants, *MEA* is specifically expressed in the central cell nucleus of stage 12 flower and in the endosperm after fertilization (**Figure 2**). In *h1* mutant, fluorescent intensity of the *pMEA::GFP* transgene was lower than that in the wild type and the number of GFP positive ovules and seeds was significantly reduced. We detected 67.1% of *h1* ovules (94 out of 140) expressing the *pMEA::GFP* transgene compared with 85.3% (262 out of 307) in wild-type at stage 12 flowers (**Table 1**). As the seeds develop, the number of *h1* mutant seeds expressing the *pMEA::GFP* transgene increased to 79.1% compared to 91.5% in WT at 1 day after pollination (DAP) and reached 94.4% at 4 DAP which was comparable with what was seen in wild-type (95.2%). The lower ratio of GFP-positive *h1* ovules suggests that the *h1* mutant ovules mature slightly slower than wild type ovules for unknown reason. However, the GFP fluorescent intensity was noticeably lower in the *h1* mutant than in wild type using the same parameter settings under the fluorescence microscope. The *pFWA::GFP* transgene also exhibited a similar expression pattern as the *pMEA::FP* transgene. In the *h1* triple mutant seeds at 1 DAP, the GFP fluorescent intensity was much lower than that in wild type and the percentage of *pFWA::GFP* expression seeds (84.5%) was less than wild type (94.6%) (**Figure S5**, **Table 1**). As the *h1* triple mutant seeds developed, nearly all seeds expressed the *pFWA : GFP* transgene, but the GFP intensity remains lower than in wild type. In summary, *h1* mutations reduce *MEA* and *FWA* gene expression in the central cell and endosperm.

**Table 1:** The effect of the *h1* mutation on the expression of *MEA* and *FWA* in the central cell and endosperm.

	Stage 12 F/nF	F%	1 DAP F/nF	F%	4 DAP F/nF	F%
*MEA* WT	262/45	85.3%	387/36	91.5%	217/11	95.2%
*MEA h1*	94/46	67.1%	165/43	79.3%	68/4	94.4%
*FWA* WT	–	–	157/9	94.6%	–	–
*FWA h1*	–	–	377/69	84.5%	–	–

F/nF represents the ratio of fluorescent and nonfluorescent ovules or seeds. F% represents the percentage of fluorescent ovules or seeds among total checked ovules or seeds. WT, wild type Col-0; *h1*, *h1* triple mutant; stage 12, flower stage 12; DAP, Days After Pollination.

### Maternal H1 loss influences DME-mediated DNA demethylation in the central cell and endosperm genome methylation

The reduction in *MEA* and *FWA* expression and the increase in DNA methylation in their maternal alleles in *h1/H1* endosperm (Rea et al., 2012) suggested that H1 might play a role in assisting DME demethylation in the central cell. If H1 is necessary for DME demethylation, the majority of canonical DME DMRs would exhibit a lack of DNA demethylation (or hypermethylation) in the endosperm. Alternatively, the reduced *MEA* and *FWA* expression seen in *h1/H1* endosperm could be due to a locus-specific effect caused by maternal *h1* mutations that makes it less favorable for DME demethylation. In this scenario, we would expect to see the majority of DME DMRs are properly demethylated, accompanied by specific loci exhibiting an increase or reduction in DNA methylation. Since loss of DME results in a striking seed abortion phenotype (Choi et al., 2002), the largely normal *h1* mutant seeds (Rea et al., 2012) suggested that DME demethylation should be largely intact in these seeds, at least in the DME-regulated Polycomb Repressive Complex 2 (PRC2)-encoding genes (*i.e*., *MEA* and *FIS2*) critical for seed viability (Huh et al., 2008). To investigate the effect of *h1* mutations on DME demethylation, we carried out genome-wide bisulfite sequencing of the *h1/H1* endosperm derived from crosses between *h1* triple mutant or wild-type female (Col-0) and wild-type L*er* as the male parent. To avoid contamination of seed coat tissues, we prepared crude nuclei from the hand dissected embryo and endosperm tissues and used fluorescence activated cell sorting (FACS) to isolate pure embryo (2C and 4C) and endosperm (3C and 6C) nuclei for BS-seq library construction and sequencing (**Figure S6**). We used the Swift Bioscience’s Adapase™ technique (see Materials and Methods) that was successfully used for methylation profiling from ultra-low amounts of input genomic DNA (Luo et al., 2017; Luo et al., 2018). Methylomes from three biological replicates of *h1/H1* embryo and endosperm were generated. We used the Col-L*er* SNPs to sort and assign uniquely mapped reads to their respective parents of origin. The maternal:paternal read ratios for endosperm and embryo libraries tightly followed the expected ratios (2:1 for endosperm and 1:1 for embryo), indicating sample purity of FAC sorted nuclei with little maternal seedcoat contamination (**Table S1**). Pearson correlation coefficients were highly concordant between bio-reps (**Table S2**) and reads from the same tissue/genotype were pooled for subsequent analysis. We first looked at the methylation profiles of selected DME target genes in genome browser. Their methylation profiles are clearly lower in the *h1/H1* endosperm than that in the *h1/H1* embryo. However, the methylation levels of *FWA* and *MEA* (and to a more variable degree in *FIS2*) promoters were higher in *h1/H1* endosperm (**Figure S7**, upper panel), which is consistent with what we reported before that demethylation of the maternal alleles in these loci were less effective in the *h1/H1* endosperm (Rea et al., 2012). By contrast, *YUC10*, *SDC*, and *DRB2* exhibited a lower DNA methylation profiles in the *h1/H1* endosperm (**Figure S7**, lower panel), suggesting that maternal *h1* mutations variably influenced the methylation of certain imprinted genes.

The two polymorphic parental strains (Col x L*er*) allowed assessment of methylation difference between the two parental genomes. In wild-type endosperm, kernel density plot of fractional DNA methylation difference between the paternal L*er* and the maternal Col genome exhibited a center peak around zero and a small population of 50-bp windows toward the positive tail-end, representing localized demethylated regions hypomethylated on the maternal genome by DME (Ibarra et al., 2012) (**Figure S8A**, blue trace). The fractional methylation difference between paternal (wild-type L*er*) and maternal (*h1* Col) genomes also displayed a zero-centered peak and a more profound positive end bump, suggesting a more extended demethylation on the maternal *h1* genome (**Figure S8A**, red trace). However, the availability of SNPs between the Col-0 and L*er* genomes (~400,000 SNPs) (Hsieh et al., 2011; Ibarra et al., 2012) limits assessment of only a small fraction of the genome, we instead focused subsequent analysis at the genome-wide scale.

**Figure 3:**
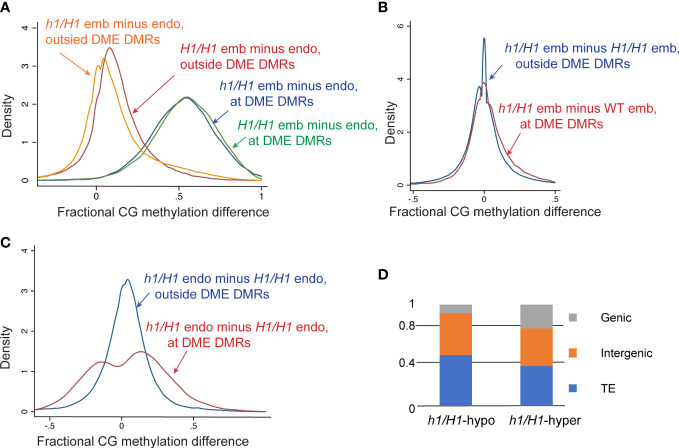
Methylome analysis of embryo and endosperm derived from the maternal wild type or *h1* mutant crossed with L*er* pollen. **(A)** Kernel density plots of CG methylation difference between embryo and endosperm (embryo minus endosperm) in wild type *H1/H1* (blue and magenta traces) or mutant *h1/H1* endosperm (green and orange traces). Methylated loci were grouped into canonical DME DMRs (50-bp windows, N=36281, blue and green traces) and outside DMR loci (N=338297, magenta and orange traces). **(B)** Kernel density plots of CG methylation difference between *H1/H1* and *h1/H1* embryo in DME DMRs (magenta) and non-DMR loci (blue). **(C)** Kernel density plots of CG methylation difference between *H1/H1* and *h1/H1* endosperm in DME DMRs (magenta) and non-DMR loci (blue). **(D)** Percent distribution of the three different genomic features in canonical DME DMRs that loose (*h1/H1*-hypo) or gain (*h1/H1*-hyper) in *h1/H1* endosperm compared with wild type.

Wild-type endosperm and embryo methylome comparison is one accepted proxy for deducing DME actions in the central cell because DME is not active in the egg cell (Hsieh et al., 2009). Thus, comparing the differentially methylated regions between wild-type Col-0 x L*er* embryo and endosperm (referred to as *H1/H1*-DMRs) would inform DME activity in the wild-type central cell. Likewise, comparing the DMRs of embryo and endosperm derived from *h1* mutant x L*er* (referred to as *h1/H1*-DMRs) would provide insights into how loss of H1 affects DME demethylation. Since the canonical DME DMRs (*dme* vs wt endosperm or *dme*-hyper DMRs) are well-defined (Ibarra et al., 2012), we separated the genome into loci within or outside *dme*-hyper DMRs and used kernel density estimate to compare the embryo-endosperm difference between Col x L*er* and *h1* mutant x L*er* seeds. For canonical DME DMRs, embryo and endosperm CG methylation difference in wild-type (*H1/H1*) (**Figure 3A**, blue trace) and *h1/H1* seeds (green trace) nearly overlap, indicating that the DME action is largely intact without maternal H1. For regions outside canonical DME DMRs, the embryo and endosperm difference are reduced (**Figure 3A**, orange trace, peak shifted toward zero) in *h1/H1* compared with *H1/H1* seeds (**Figure 3A**, red trace, peak is slightly toward the positive side). Since CG methylation is higher in embryo than in endosperm (Hsieh et al., 2009; Ibarra et al., 2012), this reduction indicates a slight overall CG hypermethylation outside of DME DMRs in the *h1/H1* endosperm. These features suggest that there is no large-scale change in DNA methylation profile in *h1/H1* seeds.

We next compared the difference between *h1/H1* and *H1/H1* methylomes. DNA methylation was largely unchanged between *h1/H1* and *H1/H1* embryos with the peak of fractional methylation difference sharply centered at zero (**Figure S8B**, blue trace) flanked by two minor shoulder peaks, indicating that localized minor hyper and hypo methylations exist in the *h1/H1* mutant embryo. This is also evident in whole genome methylation metaplots showing near identical CG methylation profiles between *h1/H1* and *H1/H1* embryos (**Figure S9**). By contrast, CG DNA methylation of the *h1/H1* endosperm is higher compared to *H1/H1* endosperm, with the fractional methylation difference peak shifted toward the right side and flanked by broader shoulders (**Figure S8B**, red trace). This suggests that a slight CG hypermethylation occurs in the *h1/H1* endosperm. Whole genome CG metaplots revealed this hypermethylation occurs in coding sequences and to a higher degree in longer TEs (**Figure S9**). This is consistent with earlier reports that heterochromatin TEs gain DNA methylation in *h1* mutant and support the hypothesis that H1-rich heterochromatin impedes DNA methyltransferase access to the DNA templates (Zemach et al., 2013; Lyons and Zilberman, 2017). Interestingly, this heterochromatin TE hypermethylation is not observed in the *h1/H1* embryo as little change in DNA methylation is observed between *h1/H1* and *H1/H1* embryos (**Figures 3B**, **S9**).

To gain a better insight into how H1 loss affects DME demethylation, we plotted the fractional methylation difference between *h1/H1* and *H1/H1* seeds within and outside of the canonical DME DMRs. Whereas very little difference between these two groups of loci was observed in embryo (**Figure 3B**), a more widespread difference can be seen within the canonical DME DMRs in the endosperm (**Figure 3C**, red trace), indicating that the loss of H1 differentially affected demethylation at the canonical DME loci. DME DMRs more demethylated in *h1/H1* endosperm (**Figure 3C**, *h1/H1*-hypo loci, fractional methylation difference <0, left peak of red trace) are enriched for TEs (50.2% versus 39.5%) but depleted for genic sequence (8.2% versus 23.6%) (**Figure 3D**) relative to DME DMRs hypermethylated in the *h1/H1* endosperm (**Figure 3C**, *h1/H1*-hyper loci, difference >0, right peak of red trace). This suggest that without H1, certain heterochromatin loci are more demethylated, which is consistent with the model that H1-bound nucleosomes restrict accessibility of chromatin modifying enzymes (Zemach et al., 2013; Lyons and Zilberman, 2017). Why some DME loci (**Figure 3C**, *h1/H1*-hyper sites) are less demethylated in the *h1/H1* endosperm is unknown, but they are enriched for chromatin states 3 and 7, hallmark features for intragenic sequences (**Figure S10**) (Sequeira-Mendes et al., 2014). For loci outside the canonical DME DMRs, there is also a slight difference between *h1/H1* and *H1/H1* endosperm (**Figure 3C**, blue trace with peak shifted toward positive side and a broader shoulder compared with embryo plot). Thus, maternal *h1* mutant specifically affects the methylation pattern of *h1/H1* endosperm but not embryo, implicating the presence of an altered DME demethylation activity in the *h1* mutant central cell.

**Figure 4:**
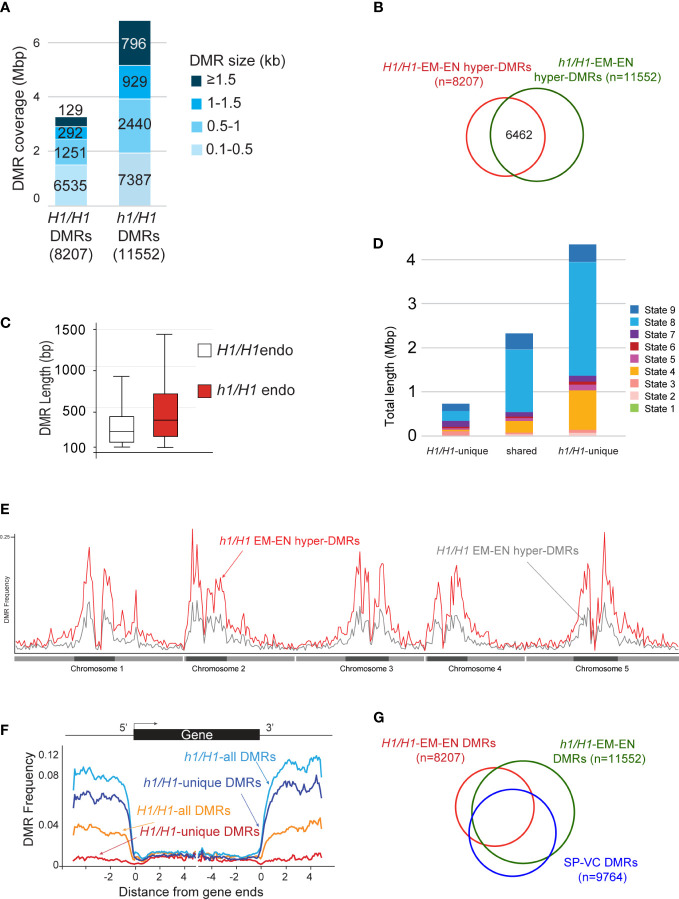
Analysis of the hyper DMRs of embryo vs endosperm derived from maternal wild type or *h1* mutant crossed with L*er* pollen. **(A)** Wild-type *H1/H1* and mutant *h1/H1* DMRs grouped by size, with the cumulative total length they cover shown. **(B)** Venn diagram depicting overlaps between *H1/H1* and *h1/H1* DMRs. **(C)** Boxplot showing the length distribution of *H1/H1* and *h1/H1* DMRs. **(D)** Chromatin state distribution, and the total length they covered, within *H1/H1*-unique, shared, and *h1/H1*-unique DMRs. States 1 to 7 correspond to euchromatin, and states 8 and 9 correspond to AT- and GC-rich heterochromatin, respectively. **(E)** Distribution frequency of DMRs along the 5 chromosomes. Dark blocks represent centromere and peri-centromeric regions of each chromosome. **(F)** Distribution frequency of DMRs with respect to coding genes. Genes were aligned at the 5’- or the 3’-end, and the proportion of genes with DMRs in each 100-bp interval is plotted. DMR distribution is shown with respect to all *H1/H1*-DMRs (orange trace), *H1/H1*-unique DMRs (red trace), all *h1/H1*-DMRs (light blue trace), and *h1/H1*-unique DMRs (dark blue trace). **(G)** Venn diagram showing overlaps between H1/H1, *h1/H1*, and sperm vs vegetative cell DMRs.

We next compared the differentially methylated regions between wild-type embryo versus endosperm (embryo hyper-DMRs in the presence of H1 or *H1/H1*-DMRs, n=8207), and between *h1/H1* embryo versus endosperm (embryo hyper-DMRs in the absence of H1 or *h1/H1*-DMRs, n=11552) (Materials and Methods) using previously established criteria (**Supplementary Dataset 1**, DMR lists) (Ibarra et al., 2012). The *h1/H1*-DMRs cover over 6 million bases, more than twice longer than the *H1/H1*-DMRs (**Figure 4A**). About 56% of the *h1/H1*-DMRs overlap with *H1/H1*-DMRs (**Figure 4B**). On average, the *h1/H1*-DMRs are significantly longer in size (**Figure 4C**, t=29.1221, *p*=0, Welch’s t-test). These observations suggest that without H1, DME demethylation is less constrained. Natural depletion of H1 in the Arabidopsis vegetative cell (VC) is associated with heterochromatin de-condensation and ectopic *H1* expression in VC impedes DME from accessing heterochromatic transposons (He et al., 2019). Consistent with this model, the *h1/H1*-unique DMRs are highly enriched for the heterochromatin states (State 8 & 9, **Figure 4D**) (Sequeira-Mendes et al., 2014). This is also supported by the DMR distribution plot across the genome showing *h1/H1*-DMRs are more abundant in the pericentromeric and genic regions (**Figures 4E, F**).

DME is also active in the vegetative cell where it demethylates a slightly larger number of loci (referred to as SP-VC DMRs, n=9764) than in the central cell (canonical DMRs, *dme* mutant versus wild-type endosperm, n=8672) (Ibarra et al., 2012). About 56% of the DME DMRs in the female gametophyte (canonical DME DMRs) and 50% of *H1/H1*-DMRs (EM vs EN, derived from Col-0 x Ler seeds) overlap with the SP-VC DMRs (Ibarra et al., 2012). One factor attributed to this moderate overlap between the male and female gametophytes is the H1-depleted, heterochromatin-decondensed vegetative nuclei that is thought to facilitate DME access to the heterochromatin targets (He et al., 2019). Notably, greater than 80% (and up to 86%) of known DME DMRs (i.e., the SP-VC DMRs, canonical DME DMRs, and WT-DMRs) overlap with the *h1/H1*-DMRs (**Figure 4G**; **Table S3**). These observations strongly support the model that lack of H1 facilitates DME access to the heterochromatin regions, which was the primary attribute for the increased number of *h1/H1*-DMRs.

### Maternal *h1* mutations do not alter gene transcription and imprinting regulation in Arabidopsis endosperm

To assess how loss of H1 influences endosperm gene transcription and imprinting regulation, we manually dissected and collected endosperm from 7-DAP F1 seeds derived from Col-0 x L*er* (*H1/H1*) and *h1* (Col-0) x L*er* (*h1/H1*) crosses. Since endosperm RNA-seq using manually dissected materials is prone to seed coat contamination (Schon and Nodine, 2017), we first tried to isolate nuclei RNAs from FACS-purified 3C and 6C endosperm nuclei. However, due to the low input amount of dissected endosperm and instability of nascent transcripts in the nuclei, we were unable to purify any detectable amounts of nuclei RNAs in our experimental setting using multiple RNA-isolation methods. We therefore proceeded with regular RNA-seq analysis using manually dissected endosperm (Hsieh et al., 2011). We used the tissue enrichment test tool (Schon and Nodine, 2017) to evaluate the extend of seedcoat contamination in our endosperm and embryo RNA-seq datasets and found that indeed a modest degree of seedcoat enrichment can be detected, with a general seed coat (GSC) enrichment score of 5 to 10 among endosperm samples and 1.8 to 3 among embryo datasets (**Figure S11**), which is consistent with, but slightly lower than, all the manually dissected endosperm datasets assessed by an earlier study (Schon and Nodine, 2017). Pearson correlation coefficients between biological replicates showed that they were highly concordant (**Table S4**). Principal component analysis showed that samples were primarily separated by tissue type, with the PC1 capturing 82% of variation while genotype difference does not differentiate tissue samples (**Figure S12**). This is also reflected in the low numbers of up- and down-regulated DEGs between *h1/H1* and *H1/H1* endosperm or embryo (fold change >2, adjusted *p* value < 0.05, 31 up and 20 down in endosperm, 5 up and 13 down in embryo, **Supplemental Dataset 2**). No GO term enrichment was detected, likely due to the small numbers of DEGs identified. RNA-seq data showed that the expression of *FIS2* and *FWA* were reduced in the *h1*-wt endosperm (with a log2 fold change of -0.41 for *FIS2* and -1.23 for *FWA*), which is consistent with what we had observed before (Rea et al., 2012). The expression of *MEA* varied between replicates but overall unchanged between wild-type and *h1* endosperm (log2 fold change 0.2). All three genes were expressed at low levels. Since histone *H1* genes are not imprinted in the endosperm (Rea et al., 2012), loss of H1 function in the gamete and central cell is likely compensated by the functional paternal copies upon fertilization. By contrast, a complete loss of H1 in the homozygous *h1* triple mutant caused a significant mis-regulation of > 900 genes in leaves and seedlings (Choi et al., 2020) and 701 gene in Arabidopsis seedlings (Rutowicz et al., 2019). Maternal depletion of H1 also did not cause TE mis-regulation (3 up- and 2 down-regulated in endosperm, 2 up- and 4 down-regulated in embryo), in contrast to the reported 18 TEs significantly dysregulated (Choi et al., 2020) and the 1.5% of TEs been mis-regulated (Rutowicz et al., 2019) in *h1* plants.

**Figure 5:**
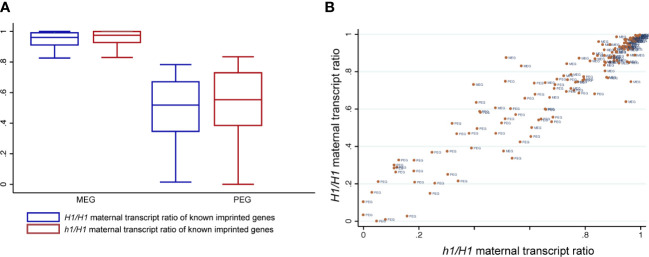
Known imprinted genes and their status of imprinted expression in *H1/H1* and *h1/H1* endosperm. **(A)** Boxplot showing the distribution of maternal transcript proportions of selected MEGs and PEGs in *H1/H1*and *h1/H1* endosperm. **(B)** Scatterplot showing the correlation of maternal transcript proportion of each imprinted gene between *H1/H1*and *h1/H1* endosperm.

We used the Col-L*er* SNPs to distinguish and sum the number of reads derived from their respective parents as we did before (Hsieh et al., 2011). To minimize mis-interpretation of possible seedcoat contamination that could skew the maternal ratio of expressed genes, we focused our analysis only on a published list of high confident imprinted gens (148 MEGs and 81 PEGs) (Schon and Nodine, 2017) and assessed any difference between *H1/H1* and *h1/H1* endosperm data that were generated by the same procedure. Among them, 80 MEGs and 42 PEGs have sufficient read numbers for proper comparison among our datasets. Overall, the maternal ratios of MEGs and PEGs are similar between *H1/H1* and *h1/H1* endosperm, with the MEGs showing a small increase in the maternal ratio (student’s t-test, P < 0.01) but not the PEGs (P > 0.05) (**Figure 5A**). Scatter plot of maternal transcript proportions among analyzed imprinted genes showed that they are highly correlated between *H1/H1* and *h1/H1* endosperm (Pearson correlation r = 0.91, n=122, **Figure 5B**) indicating that loss of maternal H1 activity does not affect overall gene imprinting regulation. Genome-wide the correlation of maternal transcript proportion between *H1/H1* and *h1/H1* endosperm is also high (r = 0.83, n=9054, **Figure S13**), consistent with the low DEGs detected between them. In summary, our results show that maternal genome *h1* mutations have a very limited effect on endosperm gene transcription or imprinted regulation.

## Discussion

DME, a multi-domain novel glycosylase that initiates active DNA demethylation process by removing the methylated cytosine bases in the central cell, regulates gene imprinting and is essential for seed development in *Arabidopsis* (Choi et al., 2002; Gehring et al., 2006; Ibarra et al., 2012). In pollen, DME also demethylates the VC genome at thousands of loci to reinforce gamete TE silencing and to ensure robust pollen germination in certain accessions (Schoft et al., 2011; Ibarra et al., 2012). DME preferentially influences small and genic-flanking transposons whose demethylation affects the transcription of nearby coding genes, it also demethylates many intergenic and heterochromatin sequences. How DME is specifically recruited to these loci remain elusive, due to its ephemeral expression in the gamete companion cells not accessible by common experimental methods. Using a yeast two-hybrid screen, Rea et al. identified linker histone H1.2 as a candidate DME-interacting protein and demonstrated that they did physically interact in an *in vitro* pull-down assay (Rea et al., 2012). Furthermore, in the female *h1* triple mutant (*h1.1, h1.2-1, h1.3*) x wild-type L*er* endosperm, the expression of selected DME-regulated imprinted genes (*MEA*, *FIS2*, *FWA*) were reduced and the methylation of their maternal alleles were increased, suggesting that DME demethylation might be impaired due to H1 loss on the maternal genome. However, H1 loss differentially influences DNA methylation in heterochromatic and euchromatic TEs prior to DME action (Zemach et al., 2013; Rutowicz et al., 2015), making it difficult to delineate if H1 directly or indirectly involves in DME demethylation in the central cell. Furthermore, DME and its more ubiquitous paralog ROS1 are likely recruited by and make contact with specialized local chromatin environments (Qian et al., 2012), as demonstrated by a recent study showing that ROS1 binds to all 4 histone proteins *in vitro* (Parrilla-Doblas et al., 2022). Since demethylation takes place in chromatin environment where DNA wraps around nucleosomes connected by H1 linker histones, the interaction between H1 and DME might simply reflect the obligated physical contact between them.

### H1.2 and DME are co-expressed in the central cell

In *Arabidopsis*, the reproductive phase is initiated late in adult plant with the specification of meiocyte precursors known as the spore mother cells (SMCs) that transition from vegetative to reproductive cell fate. This transition is accompanied by a temporary eviction of H1.1 and H1.2 in the megaspore mother cells, the female SMCs (She et al., 2013; She and Baroux, 2015; Ingouff et al., 2017). During male gametogenesis, both H1 variants are absent in late microspore stage, remain absent in the VC nuclei, but are present in the sperm nuclei (Hsieh et al., 2016; He et al., 2019). The absence of H1 in the VCs where DME is active indicates that H1 is not a requirement for DME demethylation, at least not in the VCs.

Although we found that the *H1.1* and *H1.2* promoters are active in the central cell where DME acts (**Figures 1**, **S1**, **S2**), it remains to be independently validated whether the H1 proteins are indeed present in the central cell. However, the fact that the FACT complex is specifically required for DME demethylation in the central cell heterochromatin but not in the VC (Frost et al., 2018) suggests that CC heterochromatin is more compact than in VC, and the presence or absence of H1 proteins could contribute to this difference. Detailed study is required to elucidate the expression dynamics of H1 proteins during female gametogenesis and throughout seed development.

### *H1* mutations did not cause a *dme*-like seed abortion phenotype

If H1 is required for normal DME function in the central cell, loss of H1 would be expected to induce certain degree of seed abortion similar to what’s seen in the *dme* mutant. We previously observed 16.7% seed abortion in self-pollinated F3 triple mutant *h1.1-1 h1.2-1 h1.3-1* plants (Rea et al., 2012). However, the *h1.2-1* allele has residual *H1.2* expression due to T-DNA insertion in the promoter region. This promoted us to find another stronger *h1.2-2* allele (see Materials and Methods). During the course of this study, many reports have used this *h1.2-2* allele for their studies (Zemach et al., 2013; Lyons and Zilberman, 2017; He et al., 2019; Choi et al., 2020). Each of the *h1* single mutants was backcrossed to WT for 4-6 time before analysis or used for generating the *h1* triple mutant. We examined seed phenotype of *h1* single, double, and triple mutants (*h1.1-1 h1.2-2 h1.3-1*) using different batches of plants. Seed abortion rates of *h1* single mutants *h1.1-1, h1.2-2, and h1.3-1* were 1.80%, 1.61%, and 1.26%, respectively, which were slightly higher than that of wild type Col-0 (0.62%) (**Table S5**). The *h1* double mutants *h1.1-1 h1.2-2* and *h1.2-2 h1.3-1* clearly have a cumulative effect on seed abortion, which reach 6.71% and 2.80% respectively. The *h1* triple mutant (*h1.1-1 h1.2-2 h1.3-1*) has the highest seed abortion rate (7.35%) (**Table S5**), which shows statistically significant difference from any other *h1* single and double mutants except *h1.1-1 h1.2-2*. The statistical analysis showed that seed abortion between the single mutant *h1.1-1* and *h1.2-2* and the *h1.1h1.2* double mutant was not significant (Mann–Whitney U test), but we cannot completely rule out the potential synergistic interaction between *h1.1* and *h1.2* on seed abortion. This result suggests that both *H1.1* and *H1.2* play a role in seed development. Interestingly, we also observed a large variation of seed abortion rate in histone *h1* mutants. In the *h1* triple mutant, the spectrum of seed abortion rate in individual silique varied widely from 0% to > 40%, and with more than 61% (113 out of 184) of examined siliques carrying aborted seeds (**Table S6**). This large variation of seed abortion can reflect subtle epigenetic influence of H1 on seed development as it has now been shown that H1 affects flowering time, stomata and lateral root formation, and stress response (Rutowicz et al., 2019). This can also suggest that epigenetic effect of H1 on seed development might be easily influenced by environment or subtle plant growth conditions. Since each *h1* single mutant was backcrossed to wild-type for 4-6 times, we assumed the seed abortion phenotype was caused by the T-DNA insertion. However, we cannot unequivocally ascribe the seed phenotype to the *h1* knockout without the proof of complementation results. In summary, H1 loss likely has a subtle influence on seed development but *h1* mutants do not exhibit a *dme*-like prominent seed abortion phenotype, and H1 is not a strict requirement for DME function in the central cell.

### Maternal *h1* triple mutant affects chromatin architecture and influences DME demethylation

H1 binds to the nucleosome and the linker DNA between two adjacent nucleosomes and is required for higher-order chromatin organization; yet, despite its ubiquitous presence in the nucleus and important nuclear functions, loss of H1 has very limited effects on gene transcription and TE silencing (Rutowicz et al., 2019). H1-bound nucleosomes are thought to be natural barriers to the enzymes that modify DNA, such as DNA methyltransferases (Zemach et al., 2013; Lyons and Zilberman, 2017), DNA repair enzymes, and DNA demethylases (Frost et al., 2018; He et al., 2019). Consequently, loss of H1 would facilitate access of DNA methyltransferases to the less condensed heterochromatin and resulted in a gain in heterochromatic TEs methylation as reported in (Zemach et al., 2013). By contrast, euchromatin TEs exhibited a loss in DNA methylation in *h1* homozygous mutant, for reasons currently not fully understood (Zemach et al., 2013).

Our *h1* x L*er* endosperm methylome data supports this general model of H1 function. Our results suggest that loss of H1 in the central cell (or in the maternal genome) allowed easier access of DME, particularly to the heterochromatic regions. This is reflected in a 2x increased in the number of EMB-ENDO DMRs in *h1/H1* compared with *H1/H1* seeds (**Figures 4A, B**), and the gained (*h1/H1*-unique) DMRs are enriched for heterochromatin states (**Figure 4D**) and in the pericentromeric regions (**Figure 4E**). Since there was little change between *h1/H1* and *H1/H1* embryo methylome, we can assume the difference in endosperm methylome was caused by DME action in the *h1* central cell. Our data also suggests that H1-bound nucleosomes impede DME demethylation process and the removal of H1 resulted in a significant increase in DMR length (**Figure 4C**). This notion was supported by our earlier studies that the FACT complex is specifically needed in the H1-enriched heterochromatin target loci (Frost et al., 2018; Zhang et al., 2019). Within the canonical DME DMRs (*dme* vs wild-type endosperm), we did not observe any significant difference between *h1/H1* embryo and endosperm (**Figure 3A**, blue and green traces); but a small degree of difference is visible outside DME canonical loci with a slight hypomethylation in *h1/H1* endosperm (**Figure 3A**), consisting with gaining more DMRs in *h1/H1* due to a more extensive demethylation in the *h1* CC genomes. A direct comparison between *h1/H1* and *H1/H1* endosperm revealed that loss of H1 differentially affects DME canonical DMRs (**Figure 3C**, red trace). Canonical loci hypermethylated in *h1/H1* endosperm (**Figure 3C**, positive side of the red trace) are enriched for genic coding sequences whereas loci hypomethylated (red trace, negative side) are more enriched for intergenic and heterochromatin sequences (**Figure 3D**). Taken together, our methylome data showed the effect of H1 loss on DME demethylation most likely is indirect.

### H1 loss in the maternal genome has a very limited influence on gene transcription and imprinting regulation

Our attempt to isolate RNAs from FACS-purified endosperm nuclei was unsuccessful upon multiple trials with different RNA extraction protocols. This prevented us from performing a more detailed analysis on how loss of maternal H1 influences gene imprinting regulation. Due to the presence of maternal seedcoat tissues, revealed by tissue enrichment test (**Figure S11**), we instead limited our allele-specific expression analysis on a list of previously reported high-confident imprinted genes (Schon and Nodine, 2017) and qualitatively assessed whether maternal H1 loss induced a significant distortion on their allele-specific expression. Very few differentially expressed genes or TEs were identified between *h1/H1* and *H1/H1* in endosperm and in embryo, suggesting that the wild-type H1 alleles from pollen can quickly compensate for the loss of maternal copies upon fertilization. We used the ratio of maternal over total transcripts as a measurement of parental bias and did not identify any deviation between *h1/H1* and *H1/H1* among known imprinted genes. Overall, the maternal-bias score for each imprinted gene were highly correlated between *h1/H1* and *H1/H1*, with selected individual gene showing some variation visible on the scatter plot (**Figure 5B**). These observations showed that even with a greater degree of maternal genome demethylation in *h1/H1*, the influences on endosperm gene transcription or imprinting regulation was very limited.

We reported earlier the expression of *MEA*, *FIS2*, and *FWA* were reduced and their maternal copies were hypermethylated in *h1/H1* endosperm (Rea et al., 2012). Here we showed that the flanking sequences of these three genes are indeed hypermethylated in the *h1/H1* endosperm relative to wild type and except for *MEA* whose expression was variable in our RNA-seq datasets, the expression of *FIS2* and *FWA* was reduced in the *h1/H1* endosperm. This was independently supported by the promoter reporter constructs showing *MEA* and *FWA* expression were reduced in *h1* endosperm compared with wild type (**Figures 2**, **S5**). We believe this is due to the differential effect on DME demethylation in the absence of H1 (**Figure 3C**). However, we also observed a variable methylation change in other selected imprinted genes (**Figure S7**). The reason why maternal H1 loss caused hypermethylation in some genes but not others remains to be investigated.

## Data availability statement

The high throughput sequencing datasets reported in this study are deposited in the NCBI GEO under accession number GSE217279.

## Author contributions

RF, WX, and T-FH conceived the project; QH, Y-HH, CZ, AB, MR, HY, and CP performed the experiments; X-QZ, QH, Y-HH, WX, T-FH analyzed the data; RF, WX, and T-FH wrote the article with contributions of all the authors. All authors contributed to the article and approved the submitted version.
