# Supplementary material for: Loss of linker histone H1 in the maternal genome influences DEMETER-mediated demethylation and affects the endosperm DNA methylation landscape
Source: Front Plant Sci. 2022 Dec 22;13:1070397. doi: 10.3389/fpls.2022.1070397 (PMC9813442; doi:10.3389/fpls.2022.1070397)
Supplement: Supplementary file 1 [file DataSheet_1.pdf]

Supplementary Figures

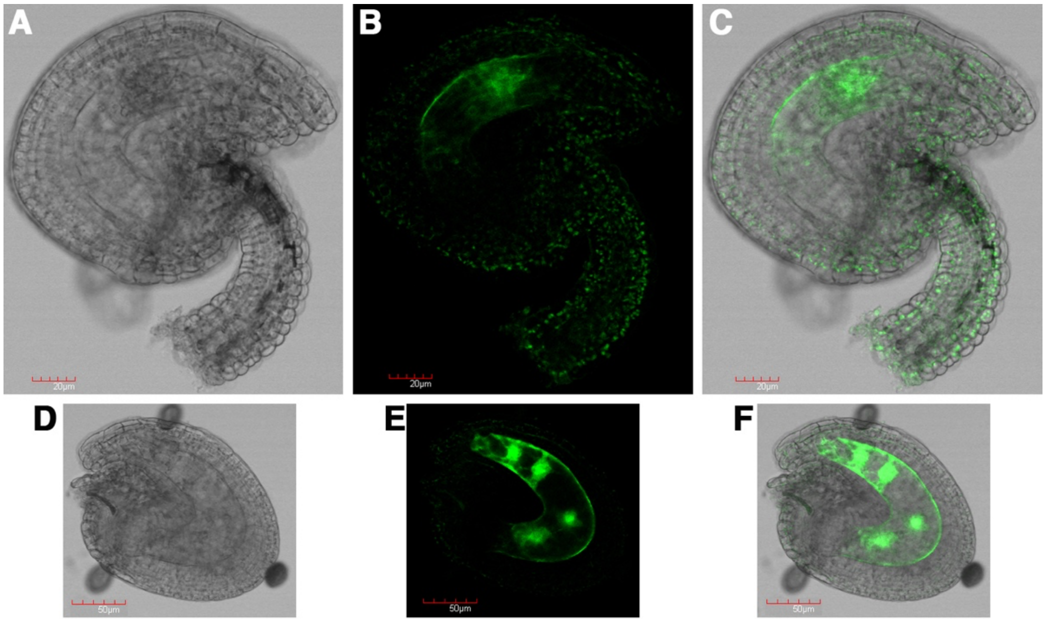

FIGURE S1. Expression of the *H1.2 promoter::GFP* transgene in *Arabidopsis* ovules and young developing seeds in different independent lines. *H1.2* expression was not visible in embryo although very weak GFP signals sometimes can be seen in the septum, funiculus, and integuments in certain lines.

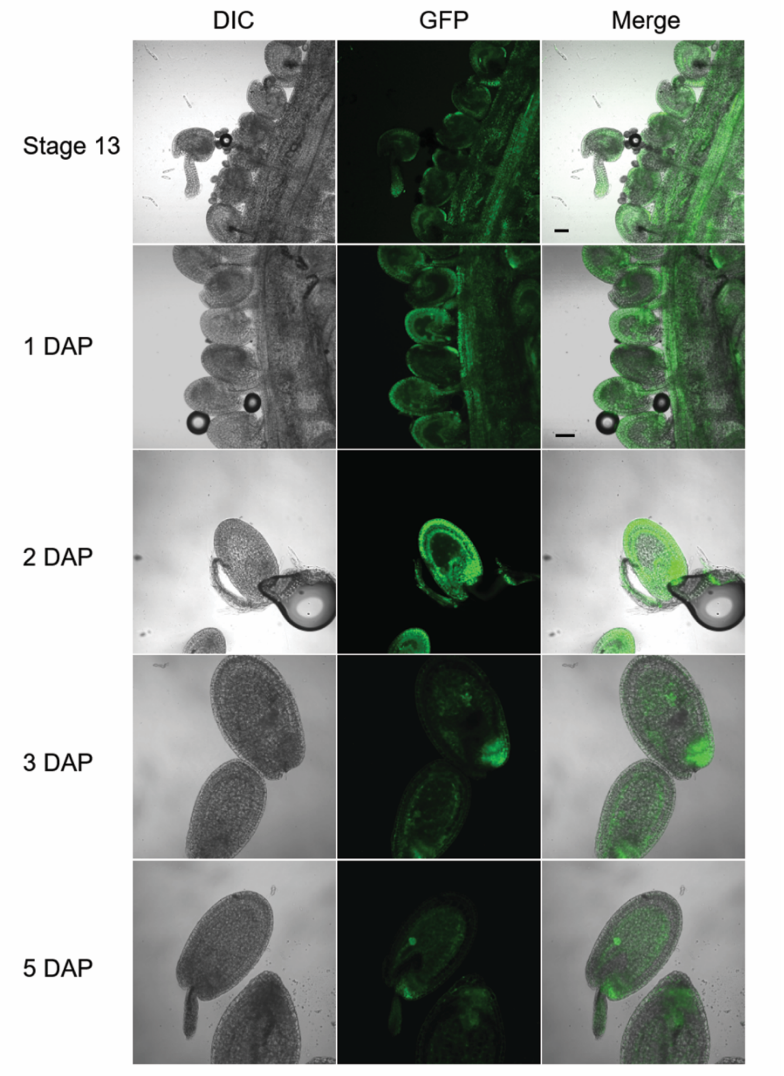

FIGURE S2. Expression of the *H1.1 promoter::GFP* transgene in *Arabidopsis* ovules and young developing seeds. Ovules and seeds with expression of *H1.1 promoter*:*GFP* transgene were photographed using confocal fluorescence microscope. Ovules at flower stage 12 and stage 13 and seeds at 1, 3, 5, and 7 DAP were hand-dissected for imaging. The GFP signal is shown in green. DAP, Days After Pollination; DIC, Differential Interference Contrast. Scale bars: 20 um at Stages 13; 50 um for 1 - 5 DAP.

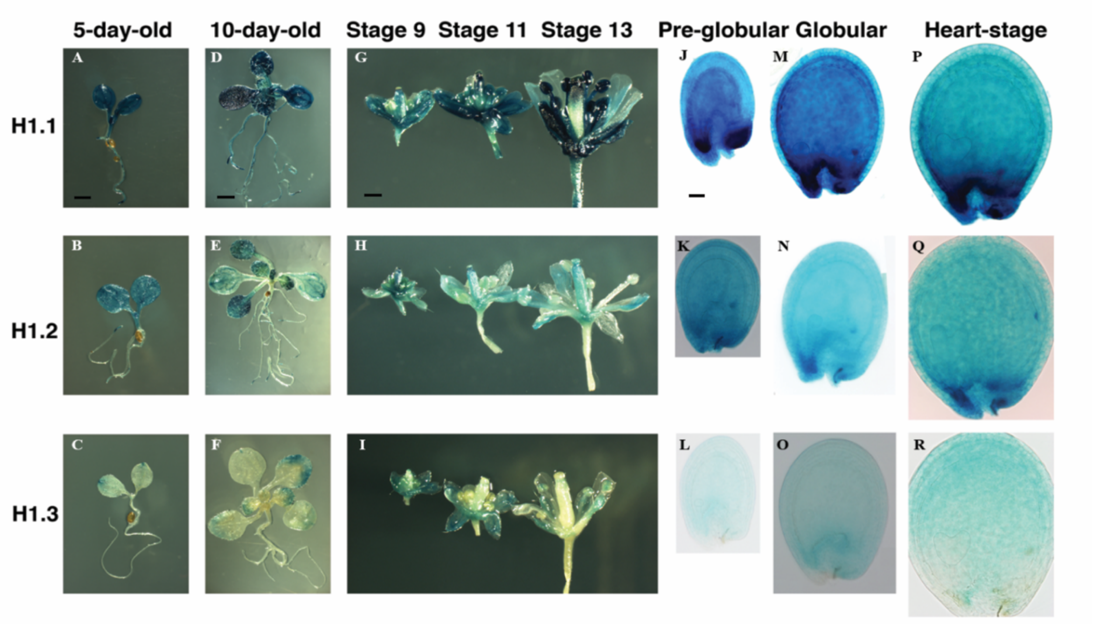

FIGURE S3. Expression of the three *H1 promoter::GUS* reporters in various *Arabidopsis* tissues. (A-C) 5-day-old young seedlings after 3-day cold treatment. (D-F) 10-day-old young seedlings after 3-day cold treatment. (G-I) Side views of opened whole flowers at stages 9, 11, and 13, respectively. (J-L) Seeds photographed when embryos are at Pre-globular stage. (M-O) Seeds photographed when embryos are at globular stage. (P-R) Seeds photographed when embryos are at Heart stage. Scale bars: A-C, 2 mm; D-F, 2 mm; G-I, 2 mm; J-R, 50 um.

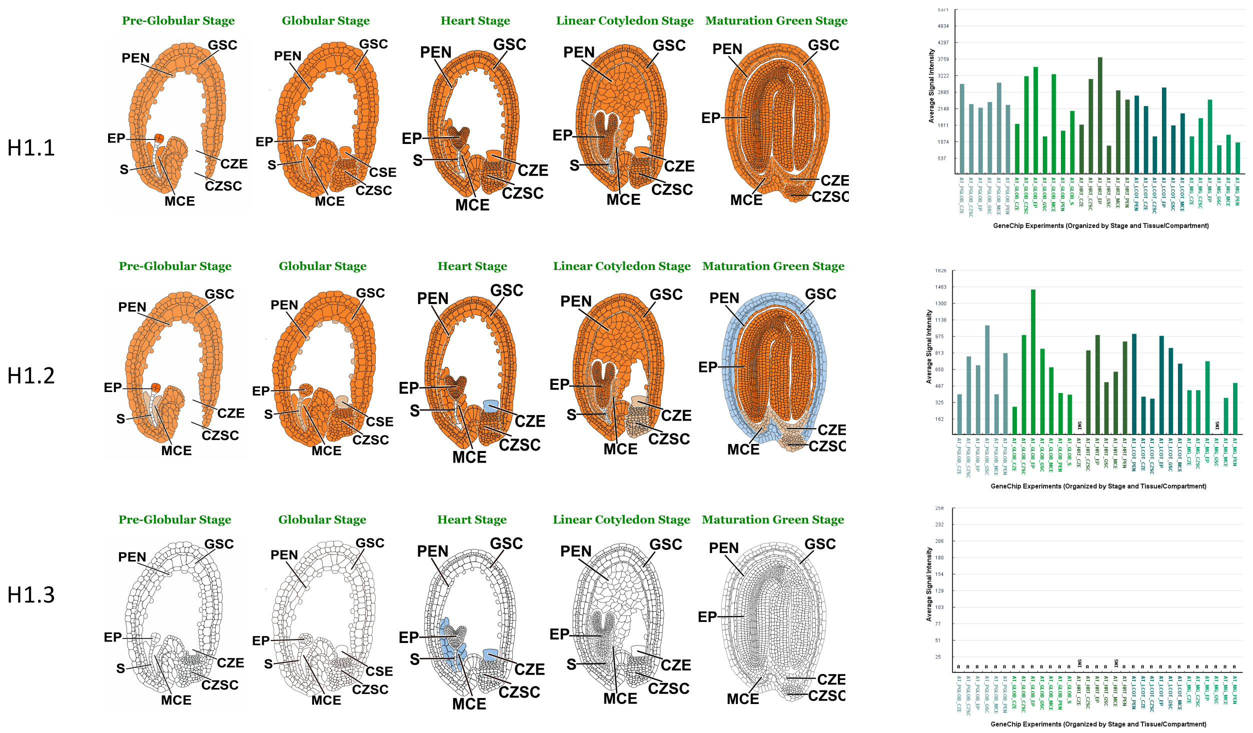

FIGURE S4. Expression of *H1.1*, *H1.2,* and *H1.3* genes during Arabidopsis seed development revealed by laser capture microdissection (LCM) and microarray analysis. Data retrieved from the Gene Networks in Seed Development database (http://seedgenenetwork.net/browse?page=1).

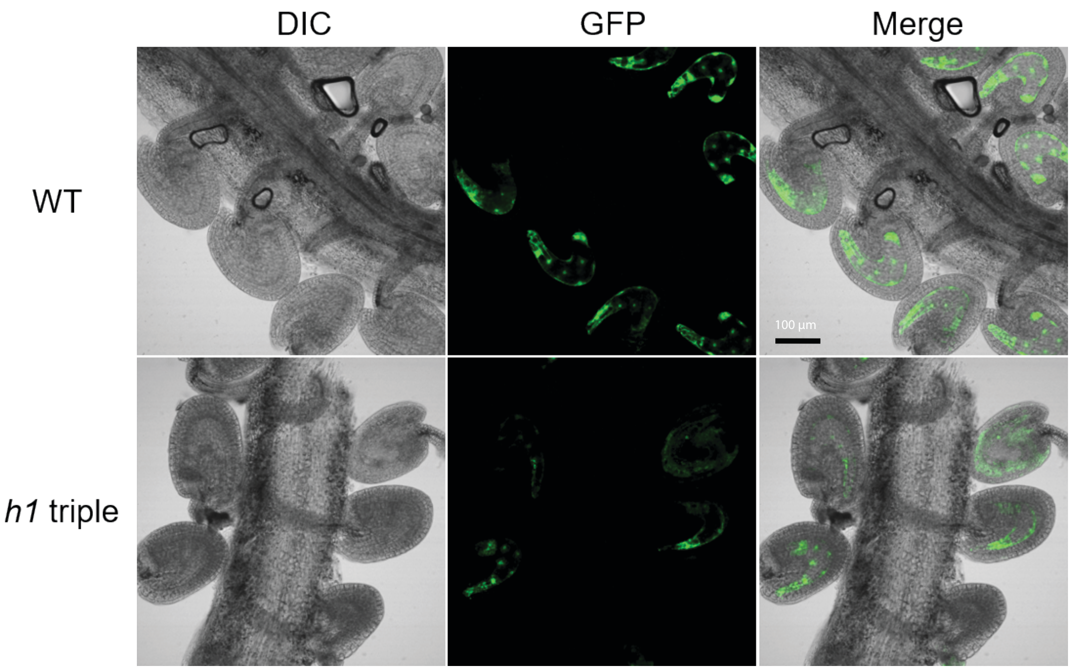

FIGURE S5. Expression of the transgene *pFWA:GFP* is reduced in the endosperm in the *h1* triple mutant. Ovules and seeds with *pFWA:GFP* transgene are photographed using confocal fluorescence microscope. Seeds at 1 DAP are dissected out for imaging. GFP expression is shown in green. DAP, Days After Pollination; DIC, Differential Interference Contrast.

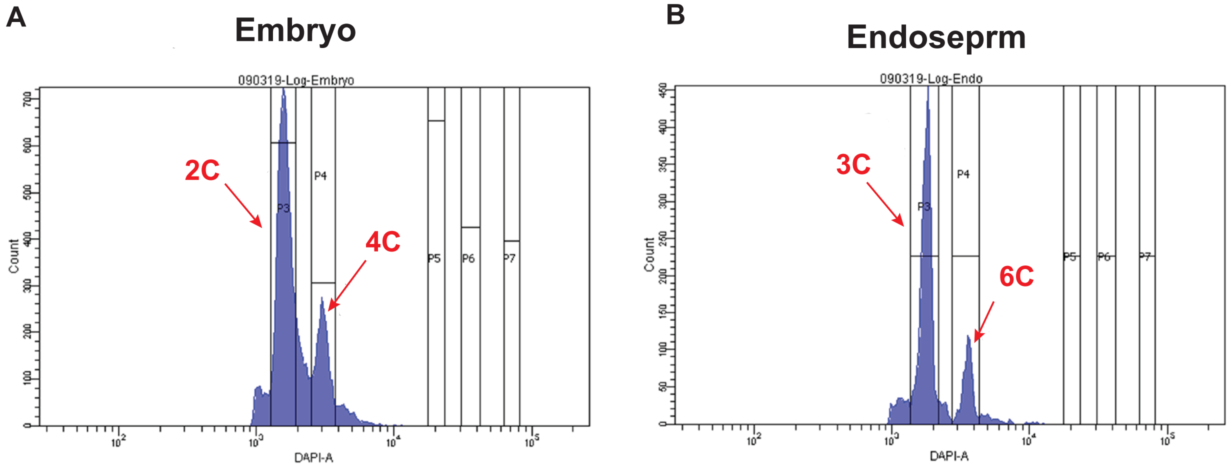

FIGURE S6. Flow cytometric profiles showing DNA contents of nuclei with different DAPI measurements. Nuclei were extracted from dissected embryo (A) and endosperm (B) derived from crosses between Col and L*er* at 7 days after pollination. The ploidy of collected peak is indicated.

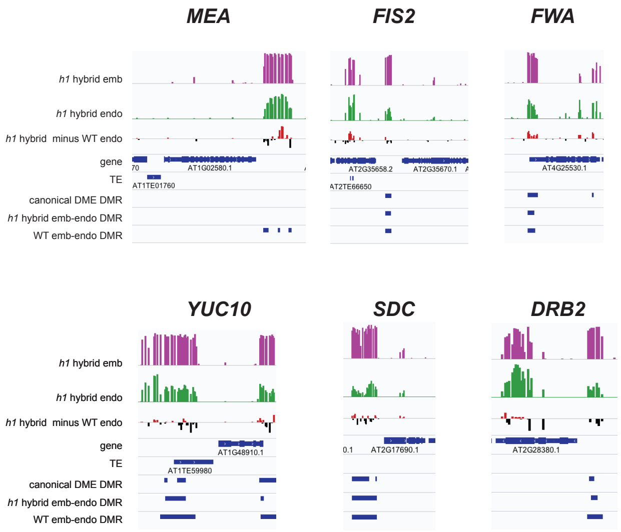

FIGURE S7. Selected imprinted genes hyper- or hypo-methylated in *h1/H1* endosperm. Genome browser snapshots of imprinted genes examples hyper- (top panel) or hypo- (bottom panel) methylated in *h1/H1* endosperm. CG methylation difference between *h1/H1* and *H1/H1* endosperm is shown in the third track. The positions of three sets of DMRs are indicated as blue boxes in the bottom three tracks.

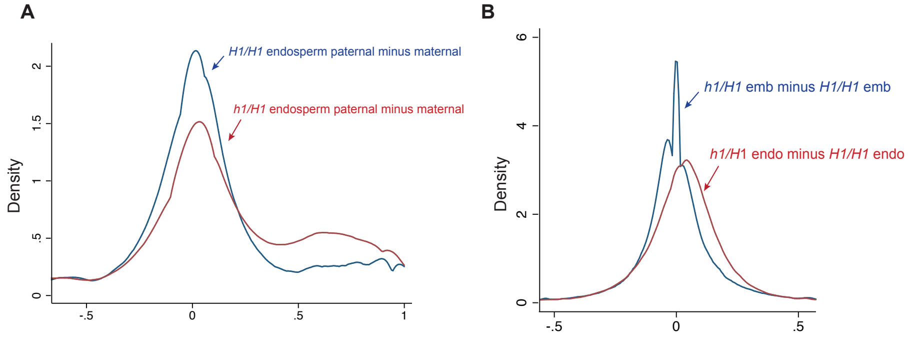

FIGURE S8. (A) Parental genome CG methylation difference in endosperm. Kernel density plot showing the differentiation in parental CG DNA methylation (paternal minus maternal) in wild type *H1/H1* (blue) and mutant *h1/H1* (red) endosperm. (B) Parental genome CG methylation difference in endosperm. Kernel density plot of CG methylation difference between *H1/H1* and *h1/H1* embryo (blue) and endosperm (magenta).

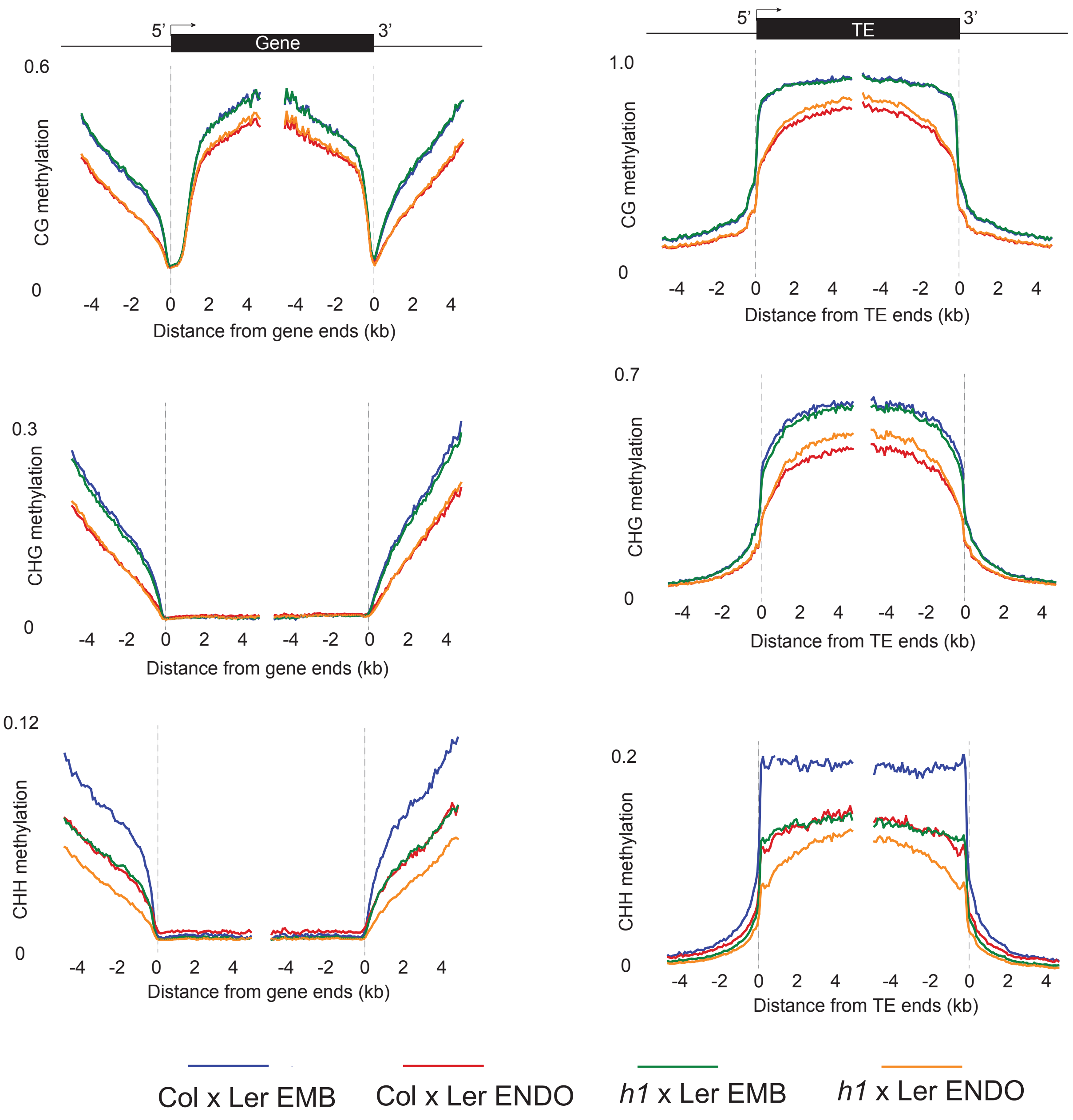

FIGURE S9. Whole genome CG, CHG, CHH methylation metaplots. Average CG, CHG, CHH methylation in Genes (left panels) or TEs (right panels) of embryo and endosperm isolated from *H1/H1* (Col x L*er*) and *h1/H1* (h1 x L*er*) seeds.

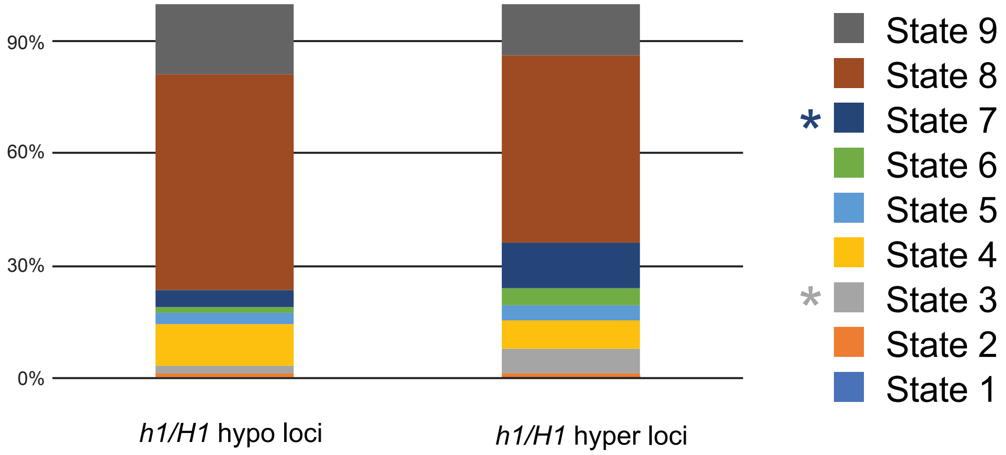

FIGURE S10. Different chromatin states in the canonical DME targets in endosperm.

Percent distribution of 9 different chromatin states in the canonical DME DMRs that loose (*h1/H1*-hypo) or gain (*h1/H1*-hyper) in *h1/H1* endosperm compared with wild type *H1/H1* endosperm.

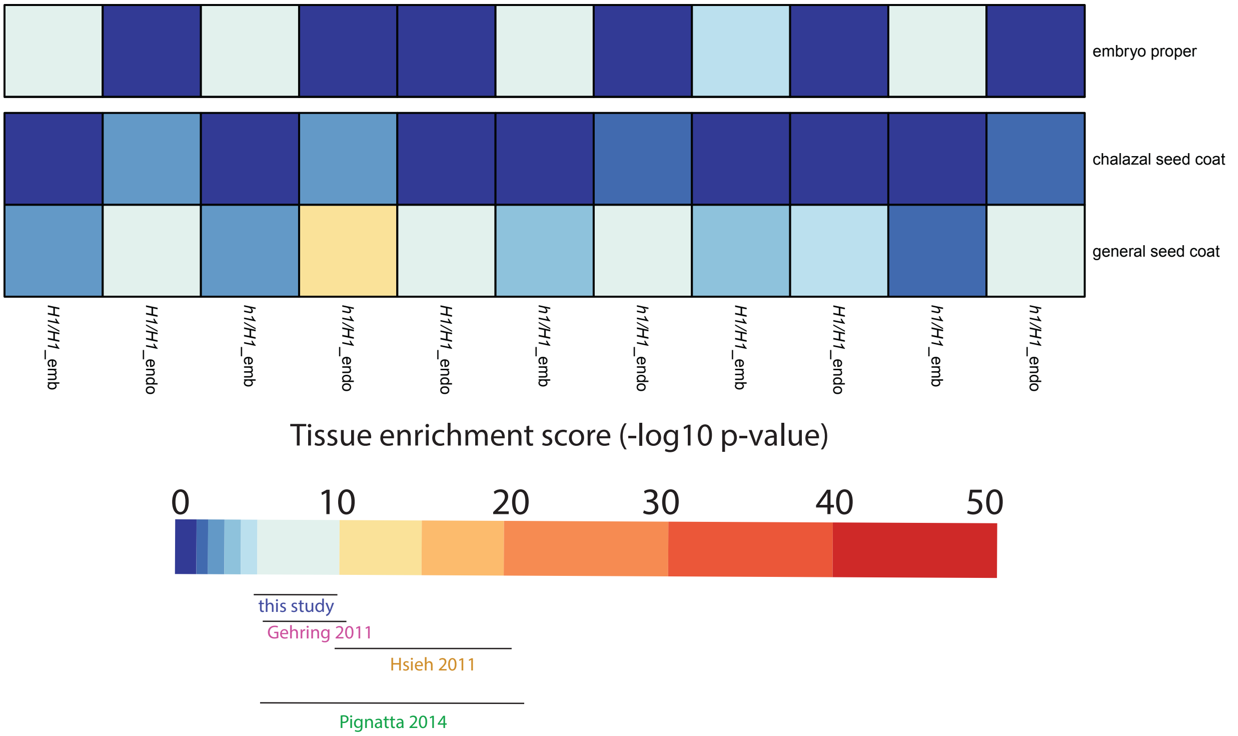

FIGURE S11. Assessing the degree of maternal RNA contamination by tissue enrichment test. Columns represent different RNA-seq transcriptomes used in this study, and rows represent two seed coat tissues. Our endosperm transcriptomes are moderately enriched for some seedcoat-specific transcripts.

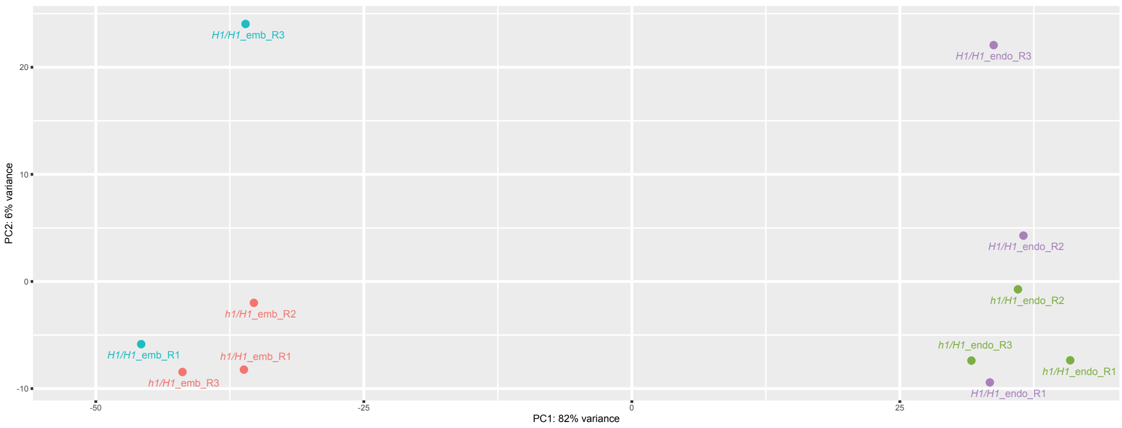

FIGURE S12. PCA analysis of all 11 samples used in this study.

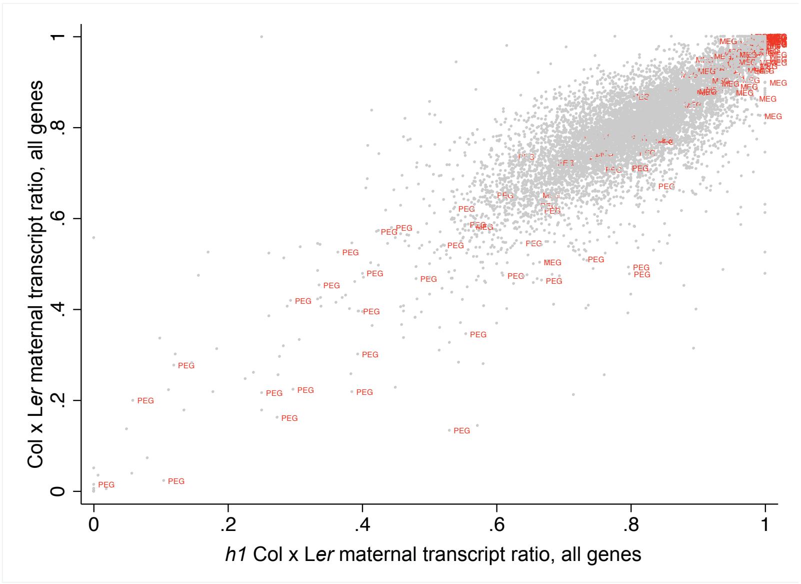

FIGURE S13. Correlation of maternal transcript proportion between *H1/H1* and *h1/H1* endosperm. Scatterplot showing the correlation of maternal transcript proportion of all genes between *H1/H1* and *h1/H1* endosperm.

Supplementary Tables

Supplemental Table 1. BS-seq dataset information

Sample	Unique mapped reads	Coverage	BS conversion rate*	M/P ratio
*h1*xLer emb rep1	12781829	7.7	99.76%	1.055
*h1*xLer endo rep1	22099703	13.3	99.74%	2.275
*h1*xLer emb rep2	35405734	21.2	99.78%	1.04
*h1*xLer emb rep3	15696292	9.4	99.66%	1.07
*h1*xLer endo rep2	39038514	23.4	99.78%	2.31
*h1*xLer endo rep3	28919583	17.4	98.82%**	2.04

*Bisulfite conversion rate was calculated using chloroplast DNA methylation ratio.
**Excluded from further analysis due to low conversion rate.

Supplemental Table 2. Whole genome *h1/H1* embryo and endosperm BS-seq data correlation between bio-replicates.

Pearson correlation coefficient	*h1/H1* emb rep1 ^{m}CG	*h1/H1* emb rep2 ^{m}CG	*h1/H1* emb rep3 ^{m}CG
h1/H1 emb rep1	1		
h1/H1 emb rep2	0.9516	1	
h1/H1 emb rep3	0.9243	0.9487	1

Pearson correlation coefficient	*h1/H1* endo rep1 ^{m}CG	*h1/H1* endo rep2 ^{m}CG	*h1/H1* endo rep3 ^{m}CG
h1/H1 endo rep1	1		
h1/H1 endo rep2	0.9563	1	
h1/H1 endo rep3	0.9157	0.9331	1

Supplemental Table 3. Pair-wise overlap between different sets of DMRs.

	SP vs VC	*dme* vs wt EN	*H1/H1*_EM_EN	*h1/H1*_EM_EN
SP vs VC	100%			
dme vs wt EN	51%	100%		
*H1/H1*_EM_EN	34%	56%	100%	
*h1/H1*_EM_EN	80%	84%	86%	100%

Supplemental Table 4. RNA-seq data correlation between bio-replicates.

Pearson correlation coefficient	*H1/H1*_endo rep1	*H1/H1*_endo rep2	*H1/H1*_endo rep3
*H1/H1*_endo rep1	1		
*H1/H1*_endo rep2	0.8355	1	
*H1/H1*_endo rep3	0.9489	0.8948	1

Pearson correlation coefficient	*H1/H1*_emb rep1	*H1/H1*_emb rep3
*H1/H1*_emb rep1	1	
*H1/H1*_emb rep3	0.6583	1

Pearson correlation coefficient	*h1/H1*_endo rep1	*h1/H1*_endo rep2	*h1/H1*_endo rep3
*h1/H1*_endo rep1	1		
*h1/H1*_endo rep2	0.9302	1	
*h1/H1*_endo rep3	0.7789	0.9321	1

Pearson correlation coefficient	*h1/H1*_emb rep1	*h1/H1*_ emb rep2	*h1/H1*_ emb rep3
*h1/H1*_ emb rep1	1		
*h1/H1*_ emb rep2	0.9778	1	
*h1/H1*_ emb rep3	0.9863	0.941	1

Table S5. Histone H1 mutation causes seed abortion.

**Genotype**	**Total Seeds**	**% Abortion**	**Total Seeds Per Silique**
**Wild Type Col-0**	3366	0.62%	52.6
**Wild Type L*er***	444	0.90%	49.3
h1.1-1	3664	1.80%	54.7
h1.2-2	1676	1.61%	40.9
h1.3-1	2530	1.26%	53.8
h1.1-1 h1.2-2	2534	6.71%	46.9
h1.1-1 h1.3-1	4372	1.88%	49.1
h1.2-2 h1.3-1	2145	2.80%	45.6
h1.1-1 h1.2-2 h1.3-1	8362	7.35%	45.4

Note: % Abortion is the percentage of aborted seeds in total examined seeds. % Good is the percentage of viable seeds in total examined seeds.

Supplemental Table S6. Distribution and variation of seed abortion among siliques in wild type and the *h1* triple mutant.

Seed abortion rate / Genotype	0%	0.1-4.9%	5-9.9%	10-19.9%	20-29.9%	30-39.9%	>40%	total
**WT Col-0**	48	14	2	0	0	0	0	64
h1.1-1 h1.2-2 h1.3-1	71	40	25	22	15	7	4	184

Note: the table shows the number of siliques with different seed abortion rates among total examined siliques. Seed abortion rate is the percentage of aborted seeds in each silique.
